# Enhanced Oral Delivery of Turmeric Extract via Spray-Dried Microparticles: Biopharmaceutical Performance and In Vitro Microbiota Modulation

**DOI:** 10.3390/ijms27156700

**Published:** 2026-07-27

**Authors:** Suwipa Ungphaiboon, Santad Wichienchot, Nattha Tampanna, Chutha Takahashi Yupanqui, Pimonsri Mittraparp-arthorn, Apichat Kaewdech, Teerapol Srichana

**Affiliations:** 1 Drug Delivery System Excellence Center, Department of Pharmaceutical Technology, Faculty of Pharmaceutical Sciences, Prince of Songkla University, Hat Yai 90110, Thailand; suwipa.u@psu.ac.th (S.U.); teerapol.s@psu.ac.th (T.S.); 2 Functional Food and Nutrition Program, Faculty of Agro-Industry, Prince of Songkla University, Hat Yai 90110, Thailand; santad.w@psu.ac.th (S.W.); chutha.s@psu.ac.th (C.T.Y.); 3 Center of Excellence in Functional Foods and Gastronomy, Faculty of Agro-Industry, Prince of Songkla University, Hat Yai 90110, Thailand; 4 Food and Nutrition Program, Faculty of Liberal Arts, Rajamangala University of Technology Srivijaya, Songkhla 90000, Thailand; nattha.t@rmutsv.ac.th; 5 Division of Biological Science, Faculty of Science, Prince of Songkla University, Hat Yai 90110, Thailand; pimonsri.m@psu.ac.th; 6 Gastroenterology and Hepatology Unit, Division of Internal Medicine, Faculty of Medicine, Prince of Songkla University, Hat Yai 90110, Thailand

**Keywords:** turmeric extract, chitosan, gastric cancer, gut microbiota, intestinal permeability, microencapsulation

## Abstract

To overcome the bioavailability limitations of turmeric, this study developed a spray-dried turmeric extract microparticle (TEM) formulation for improved oral delivery. We investigated its transport, anti-cancer properties, anti-inflammatory effects, and impact on gut microbiota. The TEM formulation successfully improved curcuminoid dissolution by approximately 40-fold and 5-fold compared with unencapsulated extract and curcumin, respectively, and demonstrated effective transepithelial transport. Notably, the TEM formulation showed twofold superior inhibition of gastric cancer cell growth compared to curcumin and significantly suppressed nitric oxide production compared to unencapsulated extract. Furthermore, fecal fermentation studies confirmed that TEMs modulate gut microbiota, with acetate as the predominant metabolite. These findings suggest that TEMs represent a promising standardized approach for managing gastrointestinal diseases.

## 1. Introduction

Turmeric (*Curcuma longa* L.) has been used for centuries in traditional medicine and as a dietary spice, particularly in Asian countries [1]. Its biological activities are largely attributed to curcuminoids and volatile oil constituents, including ar-turmerone and related sesquiterpenes [2]. Extensive preclinical studies have demonstrated the antioxidant, anti-inflammatory, antimicrobial, and anticancer properties of turmeric-derived compounds, supporting their potential application in gastrointestinal and systemic diseases [3,4,5]. Despite these promising pharmacological effects, the clinical translation of turmeric products remains limited by the poor aqueous solubility, low stability, and restricted oral bioavailability of curcuminoids.

Several formulation strategies have been developed to overcome these limitations, including nanoparticles [6,7], liposomes [8,9], solid dispersions [10], and polymer-based encapsulation systems [11,12]. Among these approaches, microencapsulation has attracted considerable interest because it can improve physicochemical stability, enhance dissolution, protect bioactive compounds from degradation, and facilitate controlled release [13]. Chitosan is particularly attractive as a carrier material due to its biocompatibility, biodegradability, mucoadhesive properties, and ability to transiently modulate epithelial barrier permeability, thereby potentially improving the intestinal absorption of encapsulated compounds [14].

Importantly, most commercially available formulations focus on purified curcumin, whereas whole turmeric extract contains both curcuminoids and volatile oils that may exert complementary or synergistic biological activities. Previous studies have shown that turmerones can enhance the cellular uptake of curcumin and contribute independently to anti-inflammatory and anticancer effects [15,16]. Consequently, preserving the natural combination of curcuminoids and volatile oil constituents may offer therapeutic advantages over isolated curcumin preparations. According to the Thai Herbal Pharmacopoeia, turmeric extract containing both fractions is recommended for gastrointestinal indications, highlighting its relevance as a clinically used herbal product [17].

We previously developed spray-dried turmeric extract microparticles composed of turmeric extract, chitosan, and mannitol [5,18]. This formulation demonstrated favorable physicochemical characteristics, enhanced mucoadhesion, and selective antimicrobial activity against gastrointestinal pathogens, including *Helicobacter pylori*, while sparing beneficial commensal bacteria. Moreover, toxicological evaluation revealed no evidence of acute toxicity, supporting its potential for oral administration. However, the biopharmaceutical behavior of this formulation, including intestinal transport and cellular uptake, has not been comprehensively characterized. Specifically, it remains unknown how the chitosan-mannitol carrier matrix modulates the simultaneous dissolution. The transepithelial transport of the co-encapsulated curcuminoids and volatile oil constituents across the intestinal barrier was addressed. In addition, its effects on gastric cancer cells, inflammatory responses, and gut microbial metabolism remain poorly understood.

The gastrointestinal tract represents a particularly relevant target for turmeric-based interventions because both the parent compounds and their microbial metabolites may contribute to biological activity. Increasing evidence suggests that interactions between dietary polyphenols and the gut microbiota influence host health through modulation of the microbial composition and the production of bioactive metabolites, including short-chain fatty acids (SCFAs) [19]. Understanding these interactions is therefore essential for evaluating the therapeutic potential of novel turmeric formulations.

Accordingly, the present study investigated the biopharmaceutical performance and biological activities of spray-dried turmeric extract microparticles (TEMs) to directly address these gaps. Specifically, we evaluated curcuminoid dissolution, transepithelial transport across intestinal epithelial cells, antiproliferative effects against gastric adenocarcinoma cells, anti-inflammatory activity in macrophages, and modulation of the gut microbiota using an in vitro fecal fermentation model. In addition, microbiota-derived metabolites, including SCFAs and other fermentation products, were characterized to provide mechanistic insights into the potential gastrointestinal benefits of this formulation.

## 2. Results

### 2.1. Spray-Dried Turmeric Extract Microparticles (TEMs)

**Figure 1:**
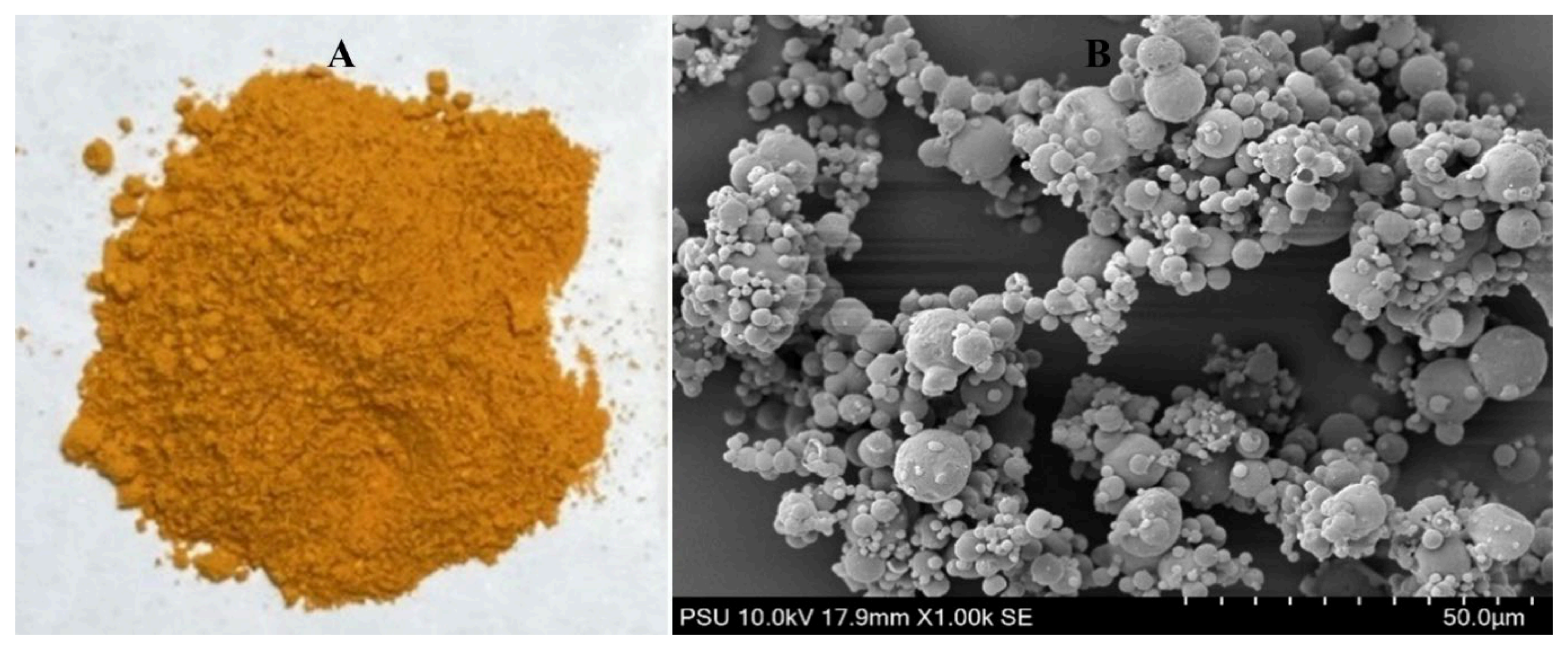
Morphology of spray-dried turmeric extract microparticles (TEMs) (**A**) and scanning electron microscope images 1000× (**B**).

Spray-dried microparticles containing standardized ethanolic turmeric extract (TEL) were prepared using a chitosan-mannitol blend as wall materials. The spray-dried turmeric extract microparticles (TEMs) appeared as an orange micronized powder with spherical shape and broad size distribution, as shown in Figure 1, and their biomarker content is presented in Table 1. The microparticles contained 24.64 ± 0.36% *w*/*w* total curcuminoids, comprising 13.09 ± 0.31% *w*/*w* curcumin, 5.17 ± 0.03% *w*/*w* demethoxycurcumin, and 5.88 ± 0.18% *w*/*w* bisdemethoxycurcumin (BDMC), together with 6.51 ± 0.04% *w*/*w* ar-turmerone. Moisture content, microbial contamination, and heavy metal levels complied with Thai FDA standards for oral herbal preparations.

Curcumin from *Curcuma longa* (CUR), a control standard, contained approximately 100% *w*/*w* total curcuminoids, while ar-turmerone was not detected. In contrast, the unencapsulated turmeric extract (TEL) exhibited the highest ar-turmerone content (9.37 ± 0.02% *w*/*w*).

### 2.2. Development of TEM Capsules for Oral Administration

Conventional turmeric capsules, which contain 250 mg of dried rhizome of *Curcuma longa* L. (equivalent to curcuminoids calculated as curcumin not less than 5% *w*/*w* and volatile oil not less than 6% *v*/*w*) are listed in Thailand’s National List of Essential Medicines and the Thai Herbal Pharmacopoeia [17]. The turmeric capsules are recommended at a dosage of 2–4 capsules taken four times daily after meals and at bedtime (100–200 mg of curcuminoids) for the relief of dyspepsia, abdominal bloating and flatulence as well as for the treatment of gastric ulcers.

The standardized micronized turmeric extract powder (TEM) was developed in hard capsule form to serve as an alternative turmeric product. The finished product complied with Thai FDA standards for oral herbal preparations, including limits for moisture content, microbial contamination, and heavy metals, as presented in Table 2. Heavy metal analysis showed that the arsenic level was below 0.05 ppm, cadmium and mercury were not detected, and lead content was low at 0.13 ppm. Microbial contamination was within acceptable limits, with total aerobic microbial count, total yeast and mold count, and bile-tolerant Gram-negative bacteria below 10 cfu/g, while *Salmonella* spp., *Escherichia coli*, and *Clostridium* spp. were absent. The capsule also exhibited satisfactory pharmaceutical characteristics, including a weight variation of 4.18%, rapid disintegration within 1.76 min, potency of 108.88% of the labeled amount, and low loss on drying (1.92% *w*/*w*), confirming compliance with the specified quality standards.

Each TEM capsule provides 50 mg of curcuminoids, allowing for a substantially reduced dosing regimen. TEM capsules require only one to two capsules administered two times daily, delivering 100–200 mg of curcuminoids per day. This formulation markedly decreases both the number of capsules per dose and dosing frequency, thereby improving patient compliance and enhancing dosing accuracy in Thai traditional medicine therapy. TEM capsules were stable after storage at 25 °C and 30 °C for 12 months, as the remaining percentage of biomarkers was more than 90% of the labeled amount.

### 2.3. Dissolution Studies and Permeation Studies Using HT-29 Cells

The dissolution and intestinal permeability of TEMs were evaluated in comparison with TEL, and CUR. The dissolution study was performed in simulated gastric fluid for 60 min using the USP Dissolution Apparatus II paddle method. TEMs showed the highest curcuminoid release in simulated gastric fluid at 60 min (36.18 ± 1.93%), which was significantly higher than TEL and CUR (*p* < 0.05), as shown in Table 3. In contrast, TEL exhibited the lowest release (0.87 ± 0.19%), while CUR showed a moderate release of 7.58 ± 0.37%. Dissolution studies in simulated gastric fluid at 60 min revealed that the microparticles increased curcuminoid release by about 40 times and 5 times compared to the unencapsulated extract and CUR.

For permeability evaluation, HT-29 human colon carcinoma cells, which contain a small proportion of mucus-secreting cells and columnar absorptive cells, were used as a permeability model system for intestinal epithelial cells (IECs) [20]. Prior to the permeability study, the cytotoxicity of the samples was assessed by measuring cellular mitochondrial activity in a concentration-dependent manner. All samples at curcuminoid concentrations of 15.63 µg/mL or lower exhibited no or negligible cytotoxicity toward HT-29 cells, with relative cell viability greater than 80%. Therefore, HT-29 cells cultured on transwell inserts were used as an in vitro gut mimic to predict intestinal absorption of the samples at a curcuminoid concentration of 15.63 µg/mL.

**Figure 2:**
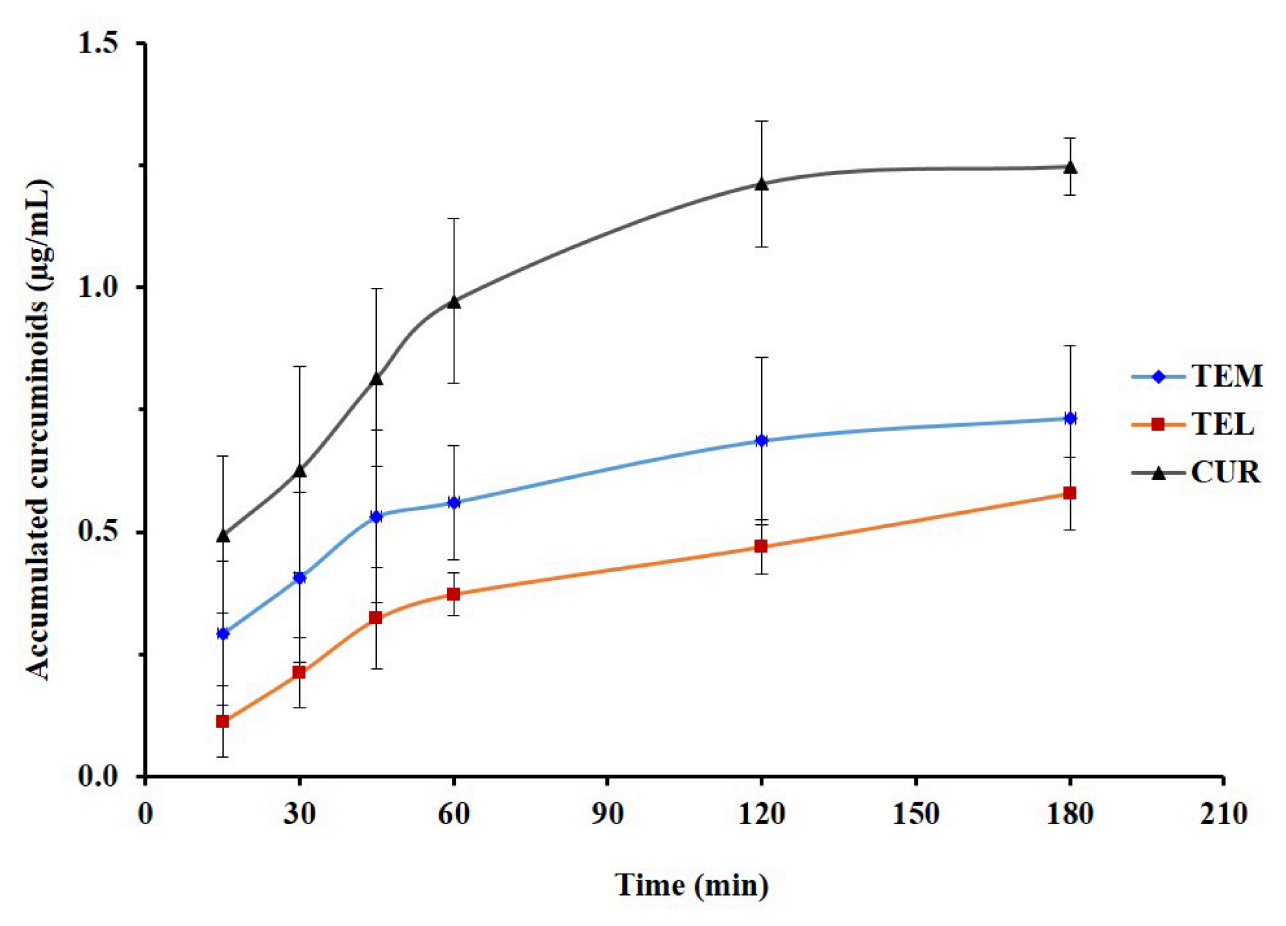
Curcuminoid flux (µg min^−1^ cm^−2^) of turmeric extract microparticles (TEM), turmeric extract in liquid form (TEL) and curcumin from *Curcuma longa* (CUR) across HT-29 monolayer (mean ± SD, *n* = 3 independent experiments).

The permeability of curcuminoids across the HT-29 model increased over time for all samples, and the Papp values were comparable among the samples up to 30 min (Figure 2 and Table 3). CUR demonstrated the highest P_app_ values throughout the transport study, reaching 17.72 ± 1.88 × 10^−6^ cm/s at 120 min and a final apparent permeability of 3.04 ± 0.14 × 10^−6^ cm/s at 180 min. TEMs and TEL showed significant lower permeability values (10.03 ± 2.52 and 6.86 ± 0.83 × 10^−6^ cm/s, respectively) at 120 min. At the end of the 180 min experiment, CUR exhibited the highest total apparent permeability, followed by TEMs (1.78 ± 0.36 × 10^−6^ cm/s) and TEL (1.41 ± 0.18 × 10^−6^ cm/s), with TEMs and TEL being significantly lower than CUR (* *p* < 0.05).

CUR showed the highest P_app_ value, while the dissolution data at 60 min was only 7.58 ± 0.37%. However, total curcuminoid content in the basolateral side was only 12% at the end of the transport experiments after 180 min. In contrast, turmeric extract in liquid form (TEL) showed the lowest P_app_ value, consistent with the dissolution data. The P_app_ of TEMs was comparable to that of CUR until 45 min. However, the transepithelial transport of curcuminoids in HT-29 cells was not significantly enhanced by the chitosan carrier, as the P_app_ of TEMs was comparable to that of the low-permeability compound TEL. Individual curcuminoids and ar-turmerone were detected, but the contents were less than the limit of quantitation by HPLC.

### 2.4. Anti-Cancer Activity on AGS Gastric Cancer Cells

**Figure 3:**
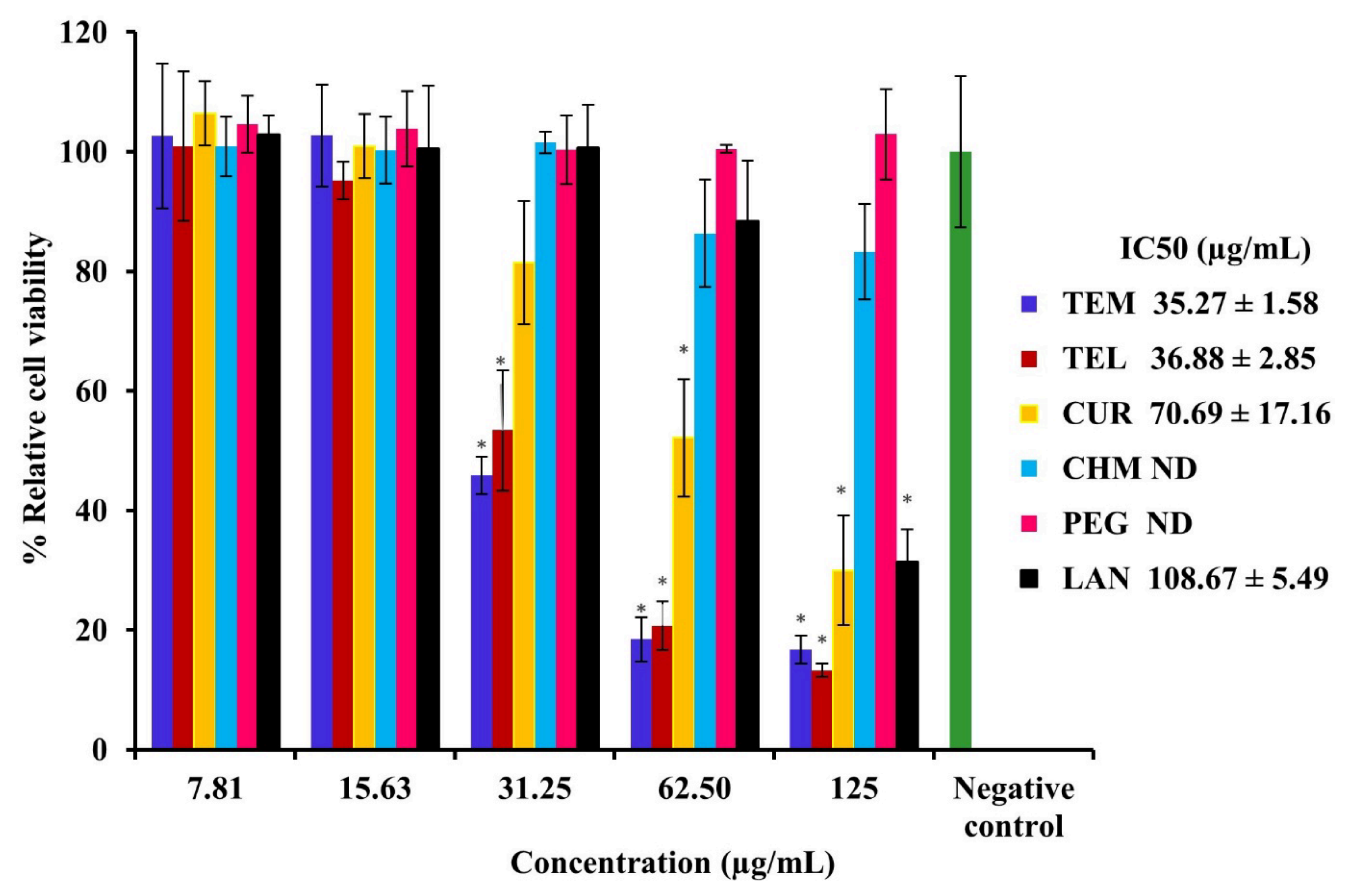
Cytotoxicity on AGS cells was detected by MTT assay. TEM = turmeric extract microparticles, TEL = turmeric extract in liquid form, CUR = curcumin from *Curcuma longa*, CHM = chitosan-based microparticles, PEG = polyethylene glycol 400, and LAN = lansoprazole. * *p*-value < 0.05 when compared with negative control (medium). Data expressed as mean ± SD, *n* = 4 independent biological replicates.

Gastric cancer is the fifth most frequently diagnosed cancer and one of the leading causes of cancer-related mortality worldwide. Influences of turmeric products and a proton pump inhibitor (PPI) drug (Lansoprazole [LAN]) on the proliferation of AGS gastric cancer cells were evaluated using the MTT [3-(4,5-dimethylthiazol-2-yl)-2,5-diphenyltetrazolium bromide] assay. After 24 h of treatment with LAN, at concentrations ranging from 8 to 63 µg/mL, AGS cell viability was comparable to that of the controls (polyethylene glycol 400 (PEG) and medium). However, starting from 125 µg/mL, a significant decrease in cell viability was observed. In contrast, TEMs and TEL induced a statistically significant reduction in cell viability compared with LAN and the controls (chitosan-based microparticles without TEL (CHMs) and PEG), as shown in Figure 3. Moreover, the viability of AGS cells treated with TEMs, TEL, and CUR decreased significantly at concentrations starting from 31 µg/mL of curcuminoids. The IC50 values, in ascending order, were as follows: TEMs (35.27 ± 1.58 µg/mL) ~ TEL (36.88 ± 2.85 µg/mL) < CUR (70.69 ± 17.16 µg/mL) < LAN (108.67 ± 5.49 µg/mL). The cytotoxicity of TEMs and TEL was comparable, but both were significantly more potent than the other two compounds (*p* < 0.05), being approximately two-fold and three-fold higher than that of CUR and LAN, respectively.

**Figure 4:**
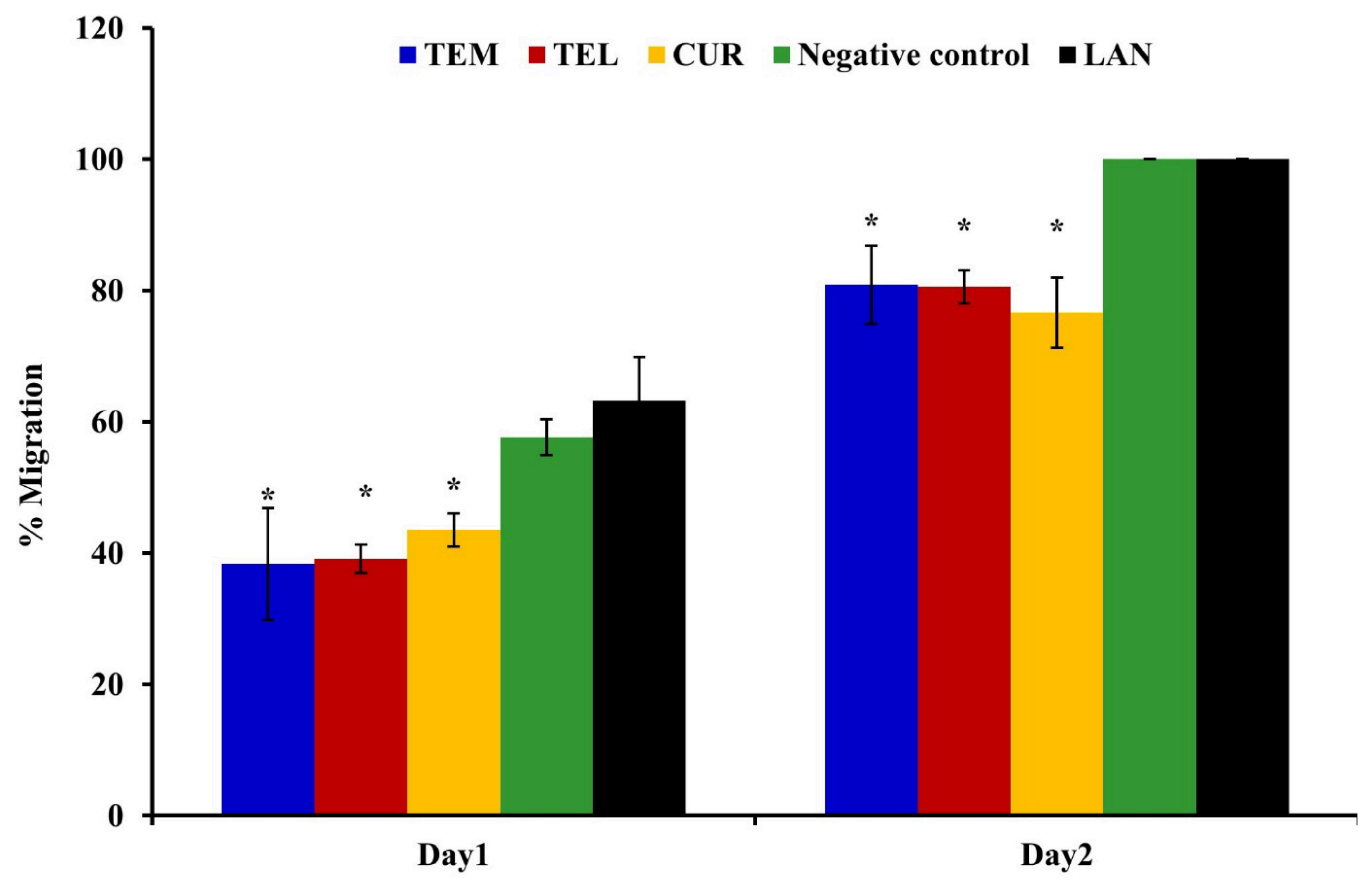
% Migration obtained from scratch wound-healing assay in AGS treated with turmeric extract microparticles (TEM), turmeric extract in liquid form (TEL), curcumin from *Curcuma longa* (CUR) and lansoprazole (LAN) at 15 µg/mL. * *p* < 0.05 when compared with negative control. Data expressed as mean ± SD, *n* = 4 independent biological replicates.

**Figure 5:**
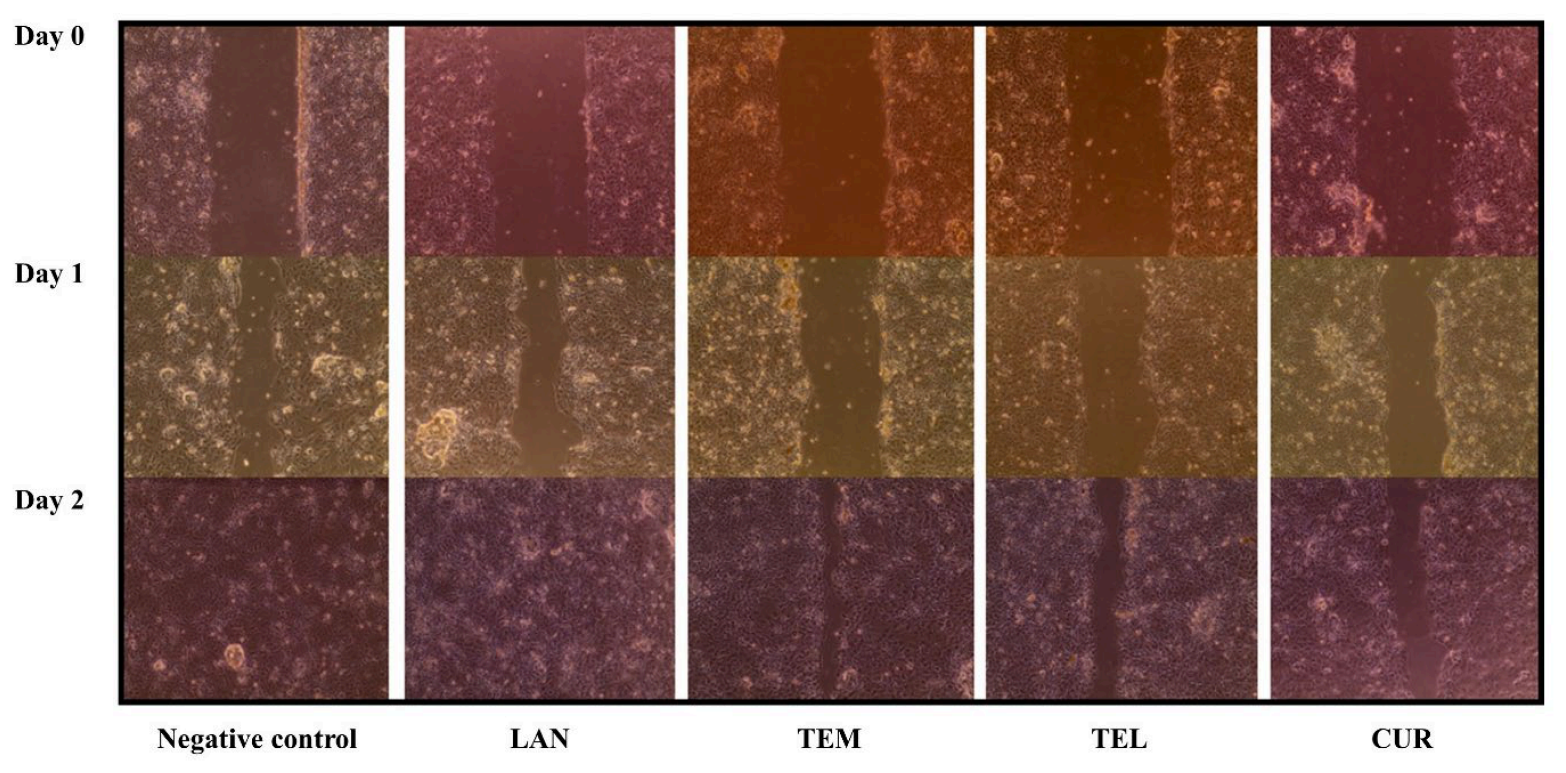
Cell migration in the in vitro scratch wound-healing assay. Stomach carcinoma cell line (AGS) layer subjected to scratch and treated with turmeric extract microparticles (TEMs), turmeric extract in liquid form (TEL), curcumin from *Curcuma longa* (CUR) and lansoprazole (LAN) at 15 µg/mL at 0, 24, and 48 h after wounding. Data are representative of four independent biological replicates (*n* = 4).

The scratch assay was performed to evaluate the cell migration ability of AGS gastric cancer cells at a sample concentration of 15.63 µg/mL, which exhibited no or negligible cytotoxicity (% relative cell viability greater than 80%). Untreated AGS cells showed a wound closure of 60% after 24 h and completely filled (100%) after 48 h of treatment, indicating that the AGS cell line has strong migratory potential (Figure 4 and Figure 5). LAN-treated AGS cells exhibited migration ability comparable to controls (medium). On the other hand, this was significantly reduced with respect to TEMs, TEL and CUR, 40% after 24 h and 80% after 48 h of treatment, as shown in Figure 4.

### 2.5. Cytotoxicity and Anti-Inflammatory Activity Studies Using Macrophage RAW 264.7 Cells

**Figure 6:**
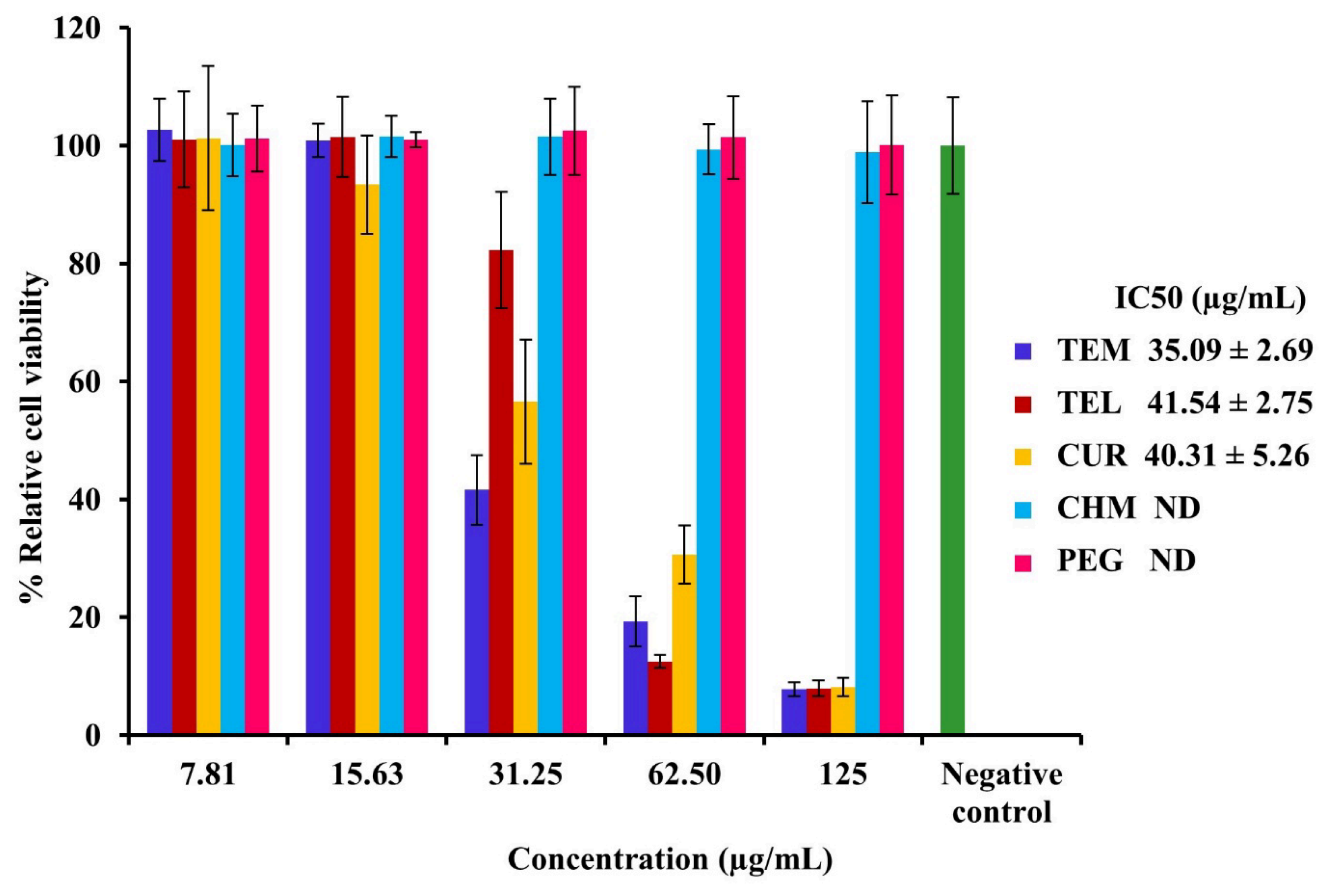
Cytotoxicity of macrophage RAW 264.7 cells. TEM = turmeric extract microparticles, TEL = turmeric extract in liquid form, CUR = curcumin from *Curcuma longa,* CHM = chitosan-based microparticles, PEG = polyethylene glycol 400 and Negative control = medium (mean ± SD, *n* = 4 independent biological replicates).

In vitro cytotoxicity testing of turmeric products was performed by measuring cellular mitochondrial activity as a function of concentration. TEMs, TEL, and CUR exhibited no or negligible cytotoxicity (% relative cell viability greater than 80%) in the macrophage RAW 264.7 cell line at curcuminoid concentrations at 15.63 µg/mL and below, as shown in Figure 6. In contrast, the formulation carriers and the solvent (PEG 400) exhibited no cytotoxicity even at the highest concentration tested (125 µg/mL). The IC50 value of TEMs was comparable to those of TEL and CUR.

**Figure 7:**
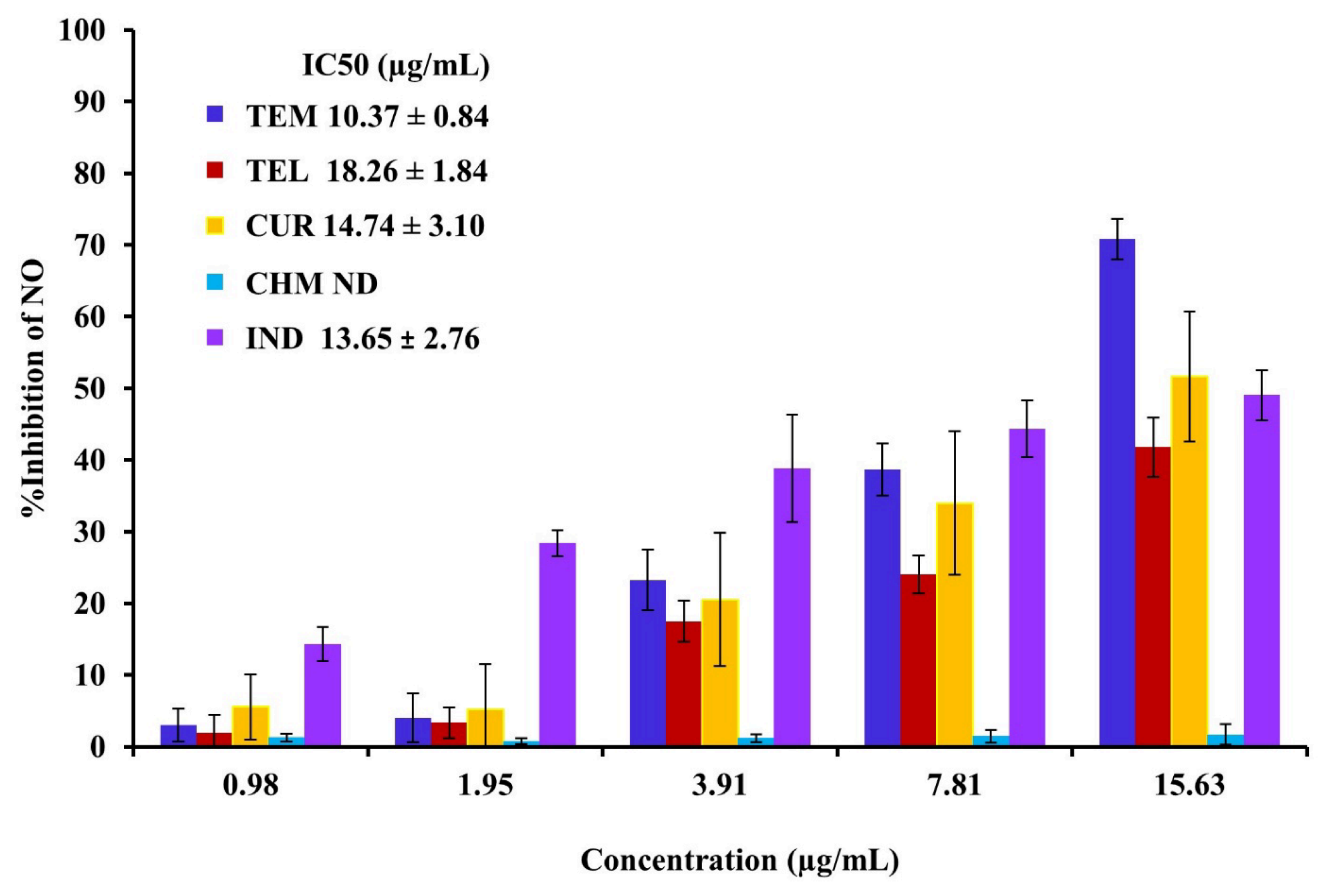
Inhibitory effect on nitric oxide (NO) production assay in macrophage RAW 264.7 cell line as a measurement of anti-inflammatory activity. TEM = turmeric extract microparticles, TEL = turmeric extract in liquid form, CUR = curcumin from *Curcuma longa,* CHM = chitosan-based microparticles, and IND = indomethacin (mean ± SD, *n* = 4 independent biological replicates).

The anti-inflammatory activity of turmeric samples was evaluated by measuring their inhibitory effect on NO production in macrophage cells. The IC50 value of TEMs was 10.37 ± 0.84 µg/mL, which was comparable to those of CUR and indomethacin. Notably, TEMs inhibited NO production in a dose-dependent manner and exhibited the highest inhibitory effect among all samples at a concentration of 15.63 µg/mL, as shown in Figure 7. In addition, TEMs showed approximately two-fold higher inhibitory activity than unencapsulated turmeric extract.

### 2.6. Gut Microbiota Modulation in a pH-Controlled Fecal Fermentation Model

#### 2.6.1. Diversity and Species Richness of Gut Microbiota

**Figure 8:**
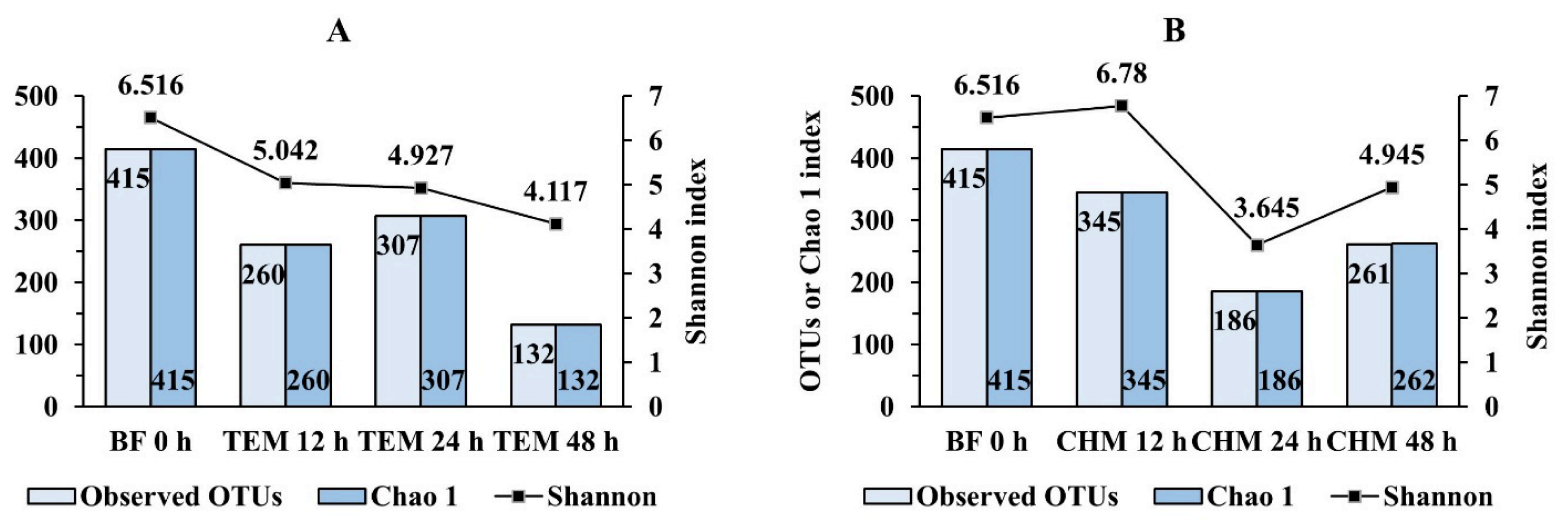
Diversity and species richness of gut microbiota after exposure to (**A**) turmeric extract microparticles (TEM) and (**B**) chitosan-based microparticles (CHM) at various fermentation times, compared to before fermentation (BF).

The diversity of bacterial populations exposed to TEMs and CHMs was assessed using alpha-diversity values at various fermentation times. Following the addition of the microparticles, a decrease in microbial diversity was observed in both groups compared to the pre-supplementation phase (before fermentation), as shown in Figure 8. Reduced alpha diversity is commonly regarded as an indicator of gut microbial dysbiosis, although its biological significance depends on the overall community composition and functional capacity. Further diversity indices, such as Chao1 and Shannon, were also calculated to provide a more comprehensive understanding of the microbial communities. The Chao1 index, which estimates species richness, showed similar trends to the observed operational taxonomic units (OTUs). The richness of bacterial species was highest in the pre-supplementation phase and lowest after 48 h of fermentation for TEMs. Within the same sample group, species richness was highest at 24 h for TEMs and at 12 h for CHMs. Species richness varied according to both fermentation time and microparticle type, indicating that both the timing and type of supplementation can significantly influence the species richness of gut microbiota. The Shannon index, which accounts for both abundance and evenness of the species present, indicated a general decrease in diversity following exposure to TEMs and CHMs. Specifically, the Shannon index values decreased from an initial value of 6.516 to a minimum of 3.645 after 24 h with CHMs, highlighting a significant reduction in microbial evenness and richness over time.

**Figure 9:**
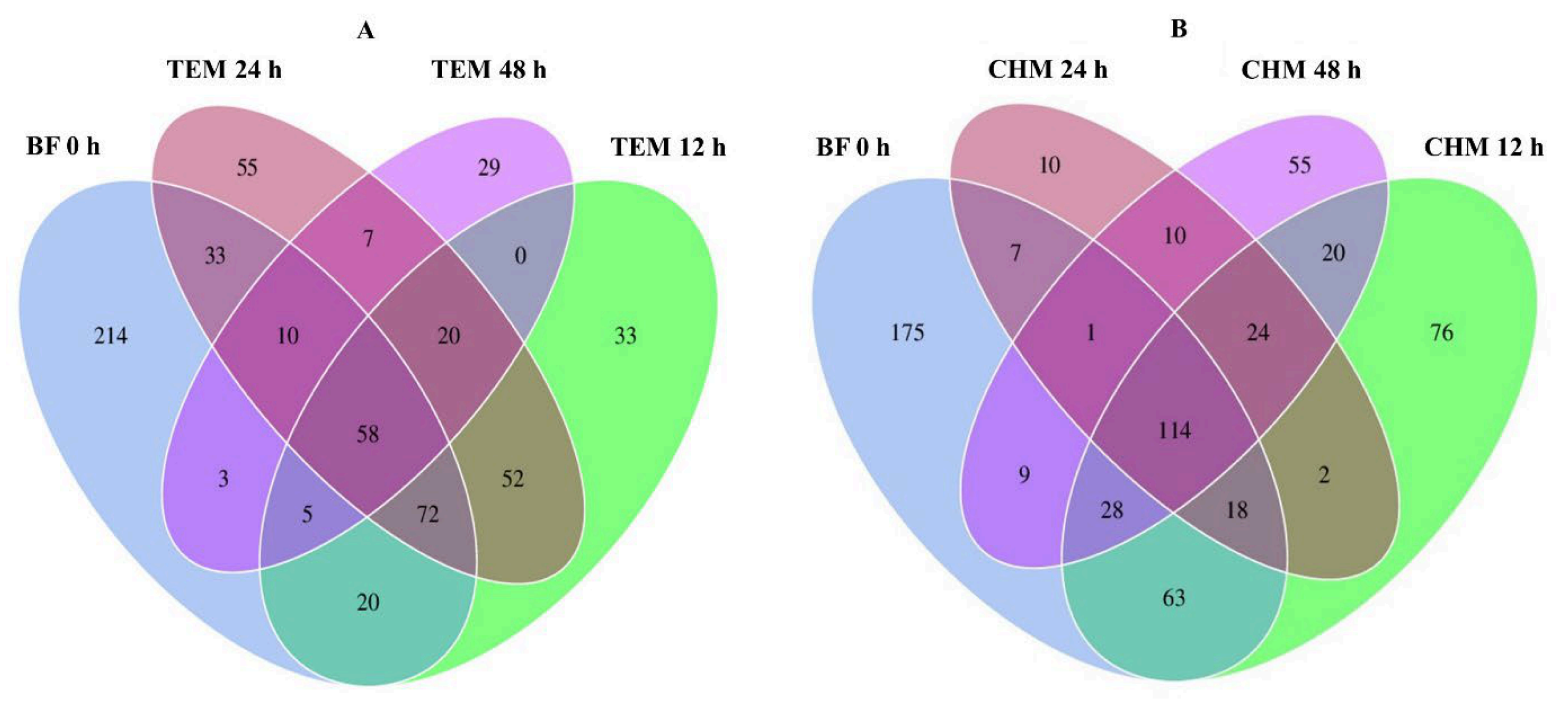
Venn diagram illustrating the number of amplicon sequence variants of microorganisms after exposed to (**A**) turmeric extract microparticles (TEM) and (**B**) chitosan-based microparticles (CHM) at various fermentation times, compared to before fermentation (BF).

The number of amplicon sequence variants (ASVs) of microorganisms in the two test sample groups at various fermentation times in the batch culture was calculated. Each circle in the figure represents a sample, with overlapping numbers indicating the common ASVs between samples, and non-overlapping numbers indicating the unique ASVs of each sample. Before the addition of the microparticles, 415 ASVs of gut bacteria were present. Upon adding TEMs and CHMs, the number of gut bacteria decreased from the initial count, as shown in Figure 9. For TEMs, specific microorganisms were identified at 12, 24, and 48 h of fermentation, with 33, 55, and 29 ASVs, respectively. There were 58 general ASVs, representing the main microorganisms commonly found in the gut that were not affected by TEMs. In contrast, the addition of CHMs resulted in specific microorganisms at 12, 24, and 48 h of fermentation, with 76, 10, and 55 ASVs, respectively and 114 general ASVs, which is higher than the number found with the addition of TEMs.

#### 2.6.2. Relative Abundance of Species

**Figure 10:**
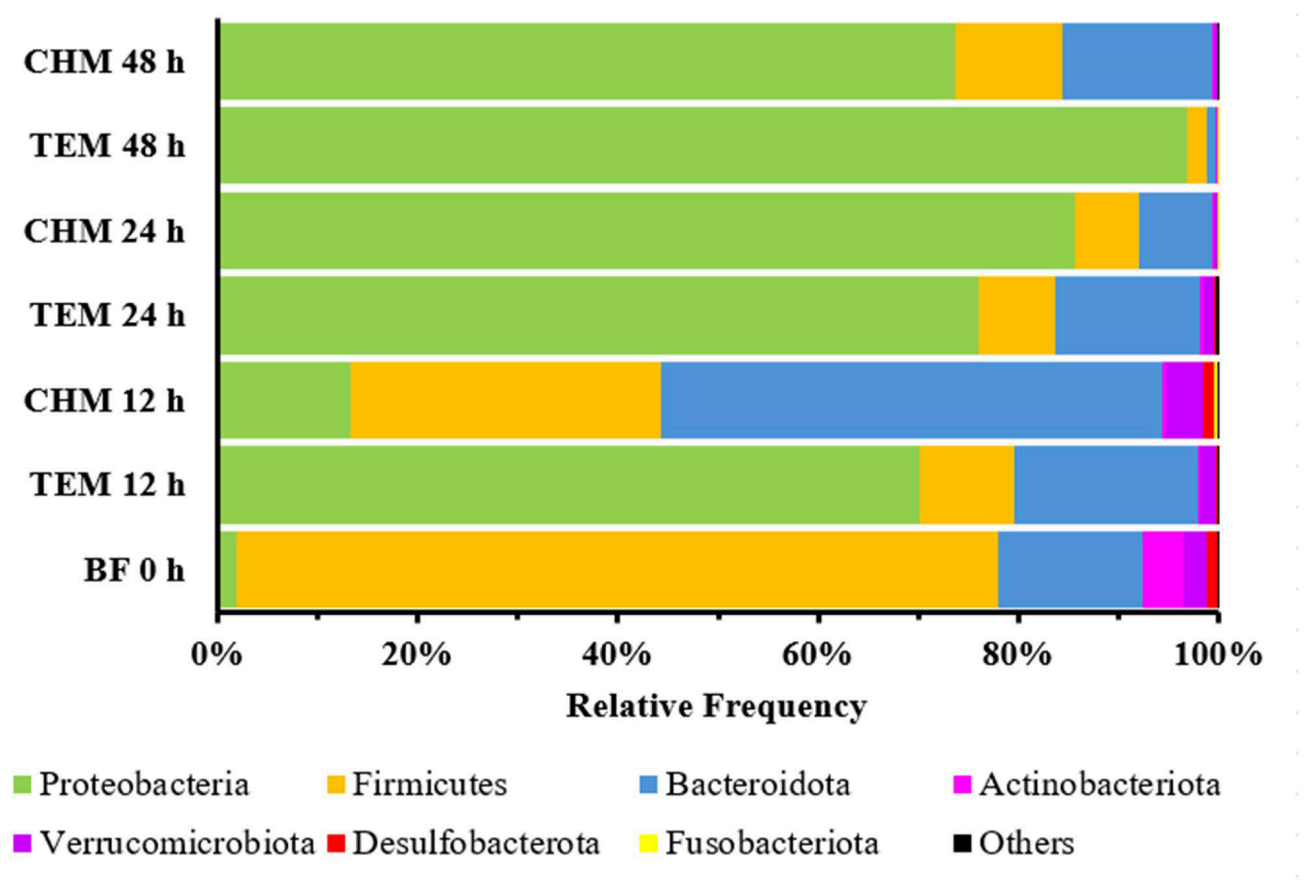
The relative abundance of bacteria at the phylum level after exposure to turmeric extract microparticles (TEM) and chitosan-based microparticles (CHM) at various fermentation times, compared to before fermentation (BF).

Histograms of the relative abundance of species were constructed to examine the species composition and proportions within each sample at different taxonomic levels. When sample sizes are large, the histograms appear dense. Abundance is represented as the mean relative abundance of the samples within each group. The analysis of bacterial abundance at the phylum level, shown in Figure 10, revealed the presence of nine phyla in the fecal samples, used as representatives of gut bacteria for analyzing changes. Before the addition of the microparticles, the most abundant phylum was *Firmicutes* (76.01%), and the least abundant was *Fusobacteria* (0.01%). Relatively few sequences were associated with the *Proteobacteria*, *Bacteroidota*, *Desulfobacteria*, *Actinobacteria*, and *Verrucomicrobia* phyla.

Following the addition of TEMs, the most abundant phylum observed was *Proteobacteria*, with relative abundances of 70.09%, 75.99%, and 96.92% at 12, 24, and 48 h of fermentation, respectively, showing an increase with longer fermentation times. The least abundant phyla were *Fusobacteria* (0.15% and 0.03%) at 12 and 24 h of fermentation, respectively, and *Verrucomicrobia* (0.01%) at 48 h of fermentation. Upon the addition of CHMs, the most abundant phylum observed was *Bacteroidota* (50.11%) at 12 h of fermentation. At 24 and 48 h of fermentation, *Proteobacteria* became the most abundant phylum, with relative abundances of 85.61% and 73.64%, respectively, showing a decrease over time. The least abundant phylum at 12 h was *Actinobacteria* (0.34%), while at 24 and 48 h, the least abundant phyla was *Fusobacteria* (0.07% and 0.02%, respectively).

**Figure 11:**
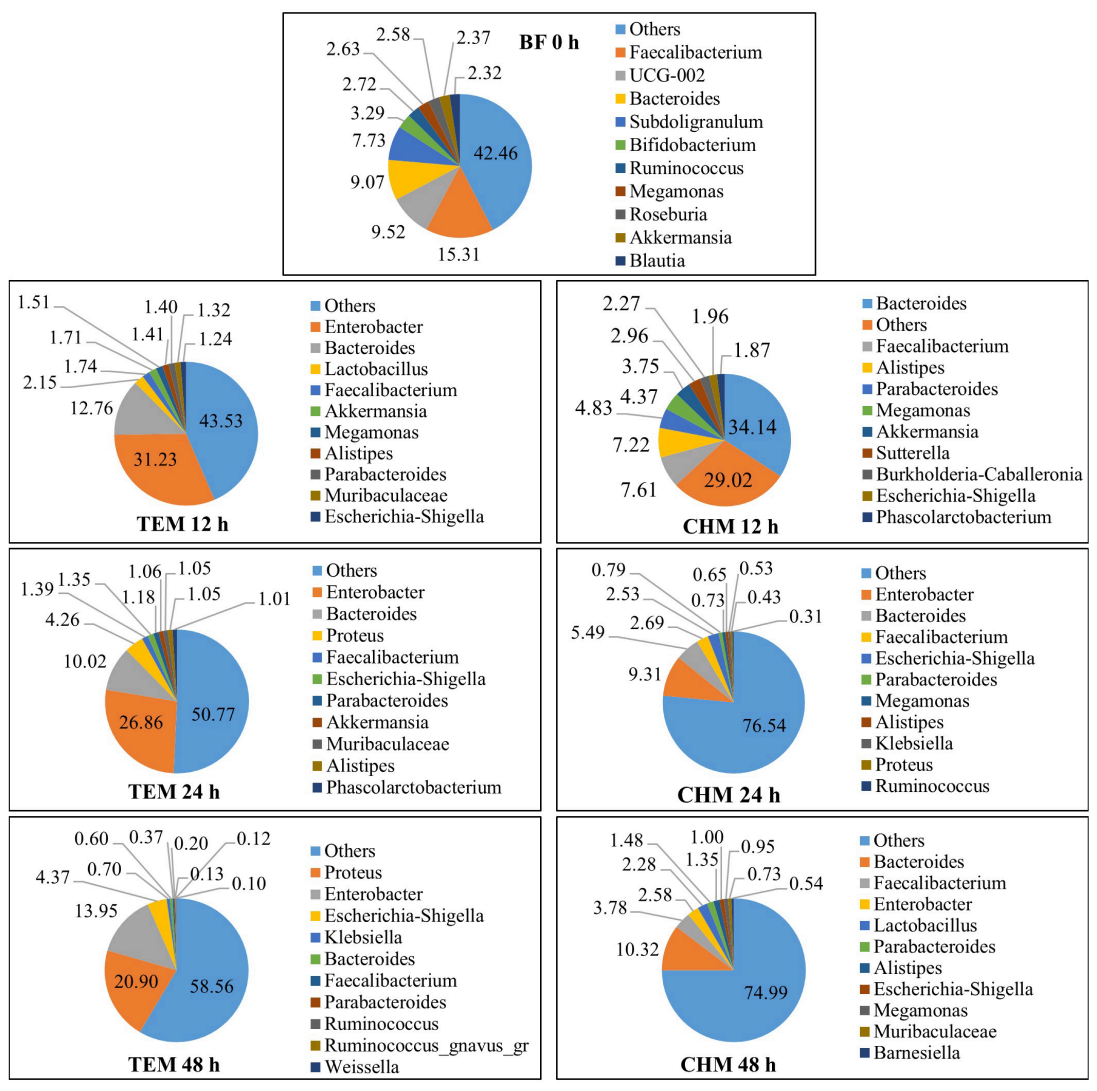
Pie charts showing the relative abundance of bacteria at the genus level after exposure to turmeric extract microparticles (TEM) and chitosan-based microparticles (CHM) at various fermentation times, compared to before fermentation (BF). Burkholderia-Caballeronia = Burkholderia-Caballeronia-Paraburkholderia.

Analysis of bacterial abundance at the genus level revealed the presence of 164 genera in the fecal samples representing the gut microbiota, with changes in abundance observed after the addition of microparticles (Figure 11). Before adding the microparticles, the most abundant genus was *Faecalibacterium* (15.31%), and the least abundant was *Parvimonas* (0.0038%). After the addition of TEMs, the most abundant genus observed was *Enterobacter*, with relative abundances of 31.23%, 26.86% and 13.95% at 12, 24, and 48 h of fermentation, respectively, showing a decrease over time. The least abundant genera were *Prevotellaceae_UCG-001*, with relative abundances of 0.0026% and 0.0013% at 12 and 24 h, respectively, and *Oxalobacter,* with a relative abundance of 0.0026% at 48 h. When CHMs were added, the most abundant genus was *Bacteroides* (34.14%) at 12 h of fermentation, decreasing at 24 and 48 h, with relative abundances of 5.49% and 10.32%, respectively. The least abundant genera were *Candidatus_Soleaferrea*, *Hydrogenoanaerobacterium*, and *Ralstonia*, with relative abundances of 0.0064%, 0.0038%, and 0.0051% at 12, 24, and 48 h, respectively.

Comparing gut bacteria at both the phylum and genus levels before and after the addition of the microparticles at various fermentation times indicated that both microparticles influence changes in the types and quantities of gut microorganisms. Following TEMs, the relative abundance of *Enterobacter* increased at 12 h and subsequently decreased at 24 and 48 h, showing more effectiveness than CHMs. After the addition of TEMs, the proportion of *Faecalibacterium* and *Akkermansia* decreased, whereas *Bacteroides* and *Enterobacter* increased at 12 h of fermentation.

**Figure 12:**
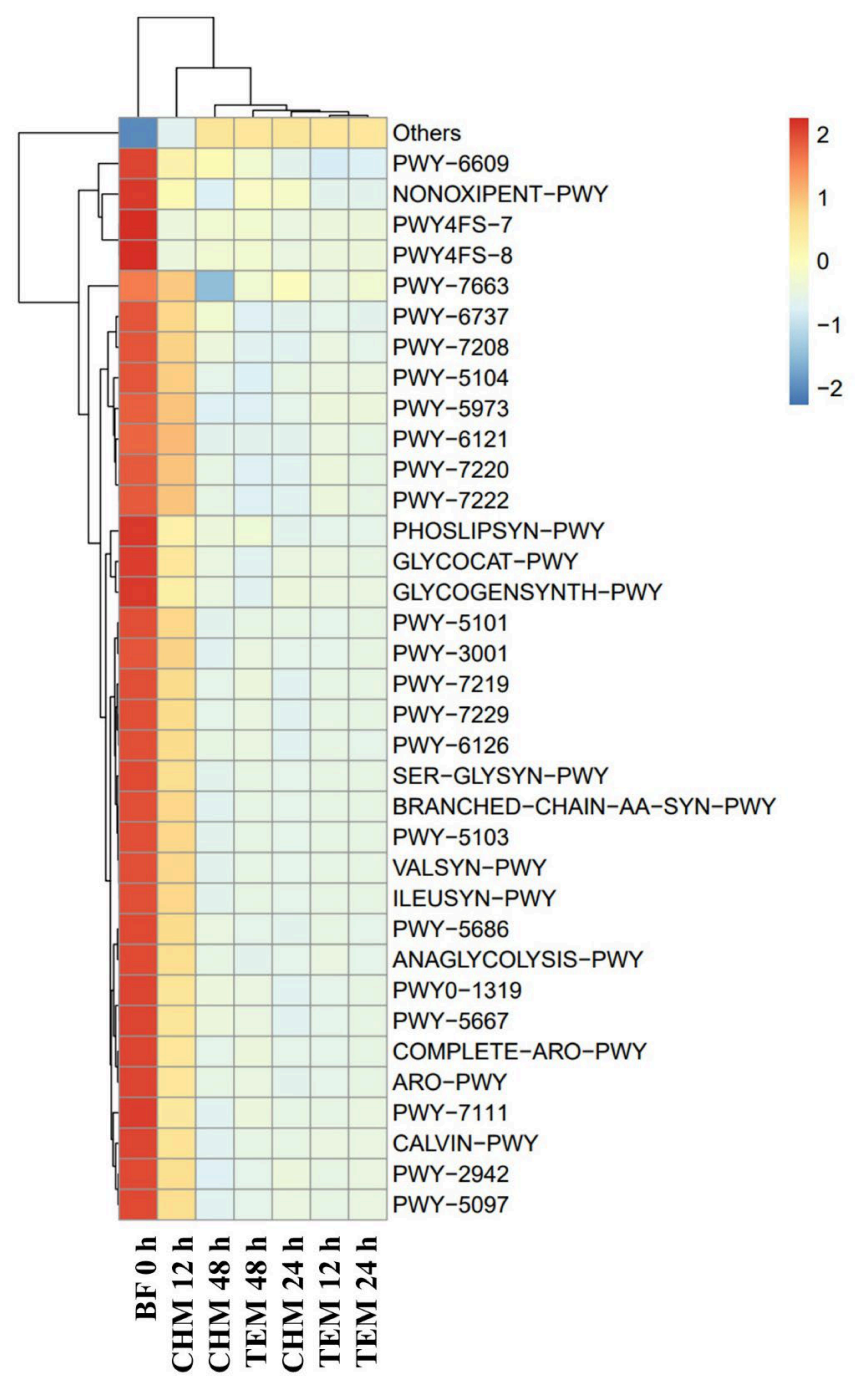
Function prediction pathways originating from microorganisms after exposure to turmeric extract microparticles (TEM) and chitosan-based microparticles (CHM) at various fermentation times, compared to before fermentation (BF).

Thirty-five functional pathways originating from the microorganisms exposed to TEMs and CHMs at various fermentation times in the fecal batch culture were predicted. The findings indicated that the highest number of predicted functional pathways was observed before the addition of test substances, with fewer predicted pathways detected after TEM and CHM supplementation, as shown in Figure 12.

#### 2.6.3. Short-Chain Fatty Acids (SCFAs)

Gut microbiota ferment dietary components that are incompletely hydrolyzed due to a lack of the appropriate enzymes, which results in the production of SCFAs, including acetate, butyrate and propionate [21]. The production of SCFAs, which are metabolites produced from the fermentation of test substances by gut microorganisms in a simulated human colon system, were analyzed and are presented in Table 4. Before adding any test substances, gut microorganisms produced all three types of SCFAs, with the highest amount of acetic acid at 2.38 ± 0.18 mM and the lowest amount of propionic acid at 0.18 ± 0.00 mM. After adding TEMs and CHMs, acetic acid increased significantly (*p* < 0.05) by approximately 3–4 times and 5–6 times, respectively, while the amount of butyric acid significantly decreased (*p* < 0.05), by about two times, with no change in propionic acid. After adding the test substances, both groups exhibited similar patterns at all fermentation times from 12 to 48 h, with acetic acid being the most abundant. After the addition of CHMs, gut microorganisms produced approximately twice as much acetic acid as observed with TEMs. When considering the same test group at different times, there was a significant (*p* < 0.05) increase in acetic acid production over time, with the highest production observed at 24 h.

#### 2.6.4. Polyphenol Metabolites

After adding TEMs (7 mM of curcuminoids), no polyphenol metabolite of curcuminoids produced by gut microorganisms in a simulated human colon system was detected. However, changes in related metabolites were observed during fermentation, as shown in Table 5. Before fermentation, L-glutamate (10.82%), γ-aminobutyric acid (GABA; 9.72%), and L-norvaline (4.38%) were the predominant metabolites. After 24 h of fermentation, TEMs were characterized by the formation of D-ornithine (20.46%), lysine (11.86%), L-valine (10.43%), and (7E)-2-acetyl-2,3,4,5-tetrahydrooxonine-6,9-dione (8.65%). In comparison, CHMs showed higher abundances of D-ornithine (38.16%), lysine (23.48%), and GABA (16.34%), with GABA increasing from 9.72% before fermentation to 16.34% after 24 h. After 48 h of fermentation, the metabolite profiles changed markedly, with the disappearance of most amino acid-related metabolites. Formylisoglutamine (17.30%) was uniquely detected in TEMs, whereas butoxyacetic acid became the predominant metabolite in both TEMs (38.80%) and CHMs (42.13%).

## 3. Discussion

This study comprehensively evaluated spray-dried TEMs as a platform formulation for the oral delivery of curcuminoids and turmerones, encompassing dissolution enhancement, intestinal transport, anti-cancer and anti-inflammatory bioactivity, and gut microbiota modulation. The results collectively demonstrate that encapsulating the whole turmeric extract rather than purified curcumin within a chitosan–mannitol matrix confers multifaceted advantages that justify the formulation strategy.

### 3.1. Dissolution Enhancement

The approximately 40-fold improvement in curcuminoid dissolution from TEMs compared with TEL in simulated gastric fluid is consistent with previous research that TEMs enhanced dissolution due to the amorphous form of curcuminoids within the microparticles, characterized by differential scanning calorimetry thermograms [5,18]. The additional five-fold improvement over commercially available curcumin (CUR) highlights the synergistic contribution of the polymeric wall system: chitosan, as a mucoadhesive cationic polysaccharide, prolongs gastric residence and disperses the microparticles, while mannitol acts as a crystallinity-disrupting excipient [22,23]. These findings align with earlier reports showing that chitosan-based encapsulation significantly enhances curcumin dissolution and reduces inter-individual pharmacokinetic variability [23,24,25].

Encapsulating the whole extract rather than purified curcumin is a strategically important distinction. The Thai Herbal Pharmacopoeia defines curcuminoid and volatile oil content standards for turmeric extract [17], and the simultaneous presence of ar-turmerone alongside curcuminoids in TEMs offers a cost-efficient multi-component product with potentially complementary bioactivities [18]. Previous spray-dried formulations of turmeric extract by our group confirmed comparable physicochemical parameters [5], and the present formulation extends those findings to a full biopharmaceutical and microbiological characterization.

### 3.2. Intestinal Permeability and HT-29 Transport Model

The permeation study using HT-29 human colonic epithelial monolayers revealed that TEMs achieved an apparent permeability coefficient (P_app_) comparable to CUR, despite substantially higher curcuminoid concentrations on the apical side. This apparent paradox can be explained by the saturation kinetics of passive transcellular transport: once apical curcuminoid concentrations exceed the transporter threshold, further increases do not proportionally augment flux [26,27]. The P_app_ of TEMs did not significantly surpass CUR, which concurs with observations by Yue et al. in Caco-2 cells, where chitosan encapsulation improved dissolution but not necessarily epithelial permeation [16]. HT-29 cells, which express both absorptive columnar cells and mucus-secreting goblet cells, constitute a closer model of the colonic environment than Caco-2 [20]; the mucus layer may additionally impede microparticulate chitosan uptake relative to free curcumin.

The transepithelial curcuminoid flux of TEMs was 1.3–1.9-fold higher than TEL at 30–180 min, suggesting that the microparticle formulation sustains dissolution, maintaining a prolonged driving force for absorption [23,28]. In addition, chitosan is known to enhance intestinal absorption by modulating tight junctions, transiently and reversibly increasing transcellular and paracellular permeability, while its mucoadhesive properties prolong residence time at the absorption site [22,24]. However, the magnitude of this enhancement appears modest in the HT-29 system, consistent with the lower chitosan concentration used here relative to microparticle formulations. Future permeation studies with differentiated Caco-2/HT-29 co-cultures incorporating a mucus layer would more closely approximate in vivo conditions.

The permeability values obtained in the HT-29 model at 180 min fall within the same range as the mean P_app_ value of 2.93 (±0.94) × 10^−6^ cm/s reported for curcumin in the Caco-2 model at 120 min [26], supporting the validity of the HT-29 system for assessing curcuminoid permeability. The absorption rates of curcumin are not uniform across concentrations (5, 10, and 20 μg/mL); absorption in the ileum decreases as curcumin concentration increases, suggesting that ileal absorption involves active transport rather than simple passive diffusion [27]. The reduced transepithelial transport observed in this study is consistent with reports of intestinal first-pass metabolism and intracellular accumulation [29]. Additionally, encapsulation within the chitosan matrix enables sustained release, increasing local drug concentration [30]. The presence of ar-turmerone in TEMs reduced curcuminoid transport into HT-29 cells over 180 min compared with CUR, which is inconsistent with findings in Caco-2 cells reported by Yue et al., suggesting cell-model-dependent differences in turmerone–curcuminoid interactions [16].

### 3.3. Anti-Cancer Activity Against AGS Gastric Cancer Cells

These findings closely align with previous studies demonstrating that the whole-extract matrix provides superior bioactivity compared to isolated compounds. Specifically, TEMs demonstrated approximately twofold superior inhibition of AGS gastric cancer cell proliferation compared with equimolar CUR, underscoring the added value of the whole-extract matrix. Turmerones, particularly ar-turmerone, have been reported to exert independent cytotoxic effects on cancer cell lines and to synergize with curcuminoids through complementary mechanistic pathways, including Mitogen-Activated Protein Kinase (MAPK) inhibition and reactive oxygen species modulation [31,32,33]. Consequently, the substantial concentration of ar-turmerone in TEMs (6.51 ± 0.04% *w*/*w*) likely drives this observed antiproliferative synergy, confirming the added value of the whole-extract matrix.

Different cancer cell types show variable sensitivities to curcumin [32]. The IC50 value reported by Wang et al. for curcumin against AGS cells (22.21 μM, 8.18 μg/mL) is lower than observed in the present study, likely reflecting differences in curcumin source, cell passage number, or solvent (DMSO vs. PEG 400) [32]. Notably, the IC50 of TEMs was 11.24 μg/mL when DMSO was used as a solvent, approximately three-fold lower than with PEG 400, confirming the solvent-dependent bioavailability effects.

Nonetheless, TEMs consistently outperformed CUR, suggesting that improved bioavailability combined with turmerone co-delivery is mechanistically relevant. Curcumin suppresses gastric cancer cell growth through the downregulation of NF-κB, inhibition of Nrf2-mediated survival signaling, and activation of apoptotic cascades [32]. Encapsulated delivery sustains intracellular curcuminoid concentrations above the apoptotic threshold for longer periods than free curcumin [34]. Scratch-wound healing data further confirmed that TEMs inhibited AGS cell migration at sub-cytotoxic concentrations (15.63 µg/mL), consistent with curcumin’s reported suppression of matrix metalloproteinase expression and epithelial-to-mesenchymal transition markers in gastric cancer [35].

The potential of TEMs as an adjunct to *H. pylori* eradication therapy is noteworthy. *H. pylori* infection is the dominant etiological factor for non-cardia gastric adenocarcinoma [36], and triple-therapy regimens including clarithromycin remain the first-line treatment in some countries. Curcumin has demonstrated bactericidal activity against *H. pylori* and enhancement of clarithromycin efficacy in vitro [35], and the improved dissolution profile of TEMs could translate into higher intragastric curcuminoid concentrations relevant to *H. pylori* eradication. The antimicrobial activity of TEMs against a range of Gram-negative and Gram-positive pathogens was previously confirmed [5], supporting this clinical hypothesis.

### 3.4. Anti-Inflammatory Activity in Macrophage RAW 264.7 Cells

The IC50 of TEMs for the inhibition of nitric oxide (NO) production in LPS-stimulated RAW 264.7 macrophages (10.37 ± 0.84 µg/mL) was comparable to CUR and superior to TEL. NO, produced by inducible nitric oxide synthase (iNOS), is a key effector of macrophage-mediated inflammation and a downstream target of NF-κB signaling [37]. Curcumin’s ability to suppress iNOS expression at the transcriptional level through NF-κB inhibition is well established [37,38]. That TEMs achieved anti-inflammatory potency comparable to pure curcumin highlights the possible contribution of turmerones and other phytochemicals in the extract, since Aggarwal et al. similarly reported that complex turmeric extracts can exhibit superior NF-κB inhibition compared with isolated curcumin fractions [38].

These findings suggest that the encapsulation of turmeric extract in chitosan–mannitol carriers enhances its anti-inflammatory potency, likely by improving curcuminoid bioavailability and enabling sustained release at the cellular level. The formulation carriers alone exhibited no anti-inflammatory activity, confirming that the observed effects are attributable to the curcuminoid payload.

### 3.5. Gut Microbiota Modulation

The pH-controlled fecal fermentation model revealed that TEMs induced pronounced alterations in gut microbial community structure. The dominance of *Proteobacteria* at 48 h (96.9%) represents a substantial compositional shift from baseline. Elevated *Proteobacteria* proportions are associated with gastrointestinal inflammation and metabolic dysbiosis [39,40]; however, whether this reflects the cytotoxic effects of high-concentration curcuminoids on anaerobic commensals or a selective growth advantage for aerotolerant *Proteobacteria* under ex vivo conditions requires further investigation. The preservation of unique amplicon sequence variants suggests a degree of community resilience despite the compositional changes.

These compositional changes are consistent with known interactions between curcumin and gut microbiota. *Escherichia coli* plays a key role in curcumin metabolism via NADPH-dependent curcumin/dihydrocurcumin reductase, converting curcumin to dihydrocurcumin and subsequently tetrahydrocurcumin [41]. Additionally, *Blautia* spp. (MRG-PMF1) can metabolize curcumin via demethylation [42], while beneficial strains including *Bifidobacterium longum*, *B. pseudocatenulatum*, *Enterococcus faecalis*, *Lactobacillus acidophilus*, and *L. casei* also possess curcumin-metabolizing capacity [43]. The selective impact of TEMs on specific microbial taxa inhibiting Gram-negative pathogens such as *Vibrio parahaemolyticus* and Gram-positive pathogens such as *Bacillus cereus*, *Listeria monocytogenes*, *Staphylococcus aureus*, and *H. pylori* [5], while not suppressing commensal *Enterobacteriaceae*, *Bifidobacterium*, or *Lactobacilli* spp., suggests that TEMs could selectively target pathogenic enteric bacteria without disrupting normal microflora. The function pathway analysis further showed a stronger fermentation correlation with CHMs than TEMs at 12 h, possibly reflecting a higher carbohydrate content in CHMs and a less pronounced impact on overall bacterial populations. The observed changes at the genus level further support the selective modulation of gut microbiota by TEMs and CHMs. In the present study, the relative abundance of *Faecalibacterium* and *Akkermensia*, genera commonly associated with butyrate production, the maintenance of intestinal barrier function, and anti-inflammatory activity [44], decreased following TEMs, whereas Enterobacter increased during the early stage of fermentation, before declining at later time point. In addition, *Bacteroides* was enriched after the addition of CHMs at 12 h. Although Enterobacter includes several opportunistic pathogenic species [45], whereas *Faecalibacterium* and *Akkermensia* are generally regarded as beneficial commensals, these taxonomic shifts should be interpreted cautiously because the physiological roles of gut microorganisms are species- and strain-dependent. Therefore, the observed changes most likely reflect selective microbial modulation by the microparticles rather than a simple increase in beneficial or harmful bacteria.

The gut microbiota changes observed here are consistent with the bidirectional interaction between curcumin and the microbiome [46]: curcumin modulates microbial composition, while microorganisms metabolize curcuminoids to bioactive products. Curcumin exhibits selective inhibitory activity against specific bacterial taxa, including pathogenic *Escherichia coli* strains, mediated partly through the inhibition of curcumin reductase and demethylase enzymes in susceptible commensals [41,42]. TEMs’ anti-*H. pylori* activity, demonstrated previously, is mechanistically relevant: preferential depletion of potentially pathogenic taxa could explain transient *Proteobacteria* enrichment through competitive release [5].

Acetate production was significantly elevated in TEM-fermented samples at 12 and 24 h relative to baseline. Acetate is the most abundant colonic SCFA and exerts systemic effects, including appetite regulation, immune priming [28,39], and lipogenesis modulation via G-protein coupled receptors [21,47]. The observed acetate increase aligns with enrichment of the acetogenic genera at early fermentation time-points and has also been noted in fermentation studies of chitosan and polyphenol-rich substrates. Butyrate, the primary energy source for colonocytes and a key regulator of intestinal barrier integrity, was not significantly elevated, which may reflect the dominance of acetogenic over butyrogenic bacteria at the tested fermentation duration [48]. A lower substrate concentration or longer adaptation period may be required to detect butyrate enhancement.

The increase in acetate production is associated with the enrichment of acetogenic genera, particularly members of the phylum *Bacteroidetes*, *Prevotella* spp., *Bifidobacterium* spp., and *Akkermansia muciniphila*. Microbial-derived acetate contributes to epithelial integrity, wound repair, and gut barrier function while reducing colonic inflammation and protecting against enteric pathogens [39]. Furthermore, acetate derived from the colonic fermentation of carbohydrates suppresses appetite via hypothalamic signaling, contributing to reduced energy intake in high-fat diet models [47]. The lower overall SCFA production with TEMs compared with CHMs likely reflects the curcuminoid-mediated inhibition of SCFA-producing bacteria, consistent with the observed decrease in *Faecalibacterium*, a key butyrate producer [48], and aligns with findings by Zhang et al. showing acetate dominance during chitosan oligosaccharide fermentation [49].

### 3.6. Polyphenol Metabolites and Amino Acid Profile

The absence of detectable curcuminoid metabolites (e.g., tetrahydrocurcumin, dihydroferulic acid) in TEM-fermented samples may reflect substrate-concentration effects: at 7 mM curcuminoids, microbial demethylation and reduction enzymes may be saturated, or the antimicrobial activity of high-dose curcumin may have suppressed the specific taxa responsible for biotransformation [41,42,50,51]. This interpretation is supported by the *Proteobacteria* dominance observed at 48 h, as curcumin-metabolizing enzymes have been characterized primarily in *Firmicutes* and *Actinobacteria* [42,46]. These findings contrast with studies reporting biotransformation at higher concentrations, where *Blautia* spp. (MRG-PMF1) converts curcumin to bis-demethylcurcumin and demethylcurcumin via demethylation at 10 mM [42], and human fecal bacteria identified 23 metabolites through acetylation, hydroxylation, reduction, and demethylation pathways at 100 μM [50].

γ-Aminobutyric acid (GABA) was detected in the CHM group, increasing from 9.72% to 16.34% after 24 h, but it was absent in TEM fermentations. This suggests that curcuminoids suppress glutamate decarboxylase activity in GABA-producing bacteria, notably *Lactobacillus* spp., *Bifidobacterium* spp., and *Bacteroides* spp. [52,53]. As an inhibitory neurotransmitter linked to the gut–brain axis, enteric GABA production has implications for anxiolytic and sleep-regulatory functions [52].

Amino acids, including lysine, L-glutamate, L-norvaline, and D-ornithine, were more abundant in CHM than TEM fermentations, consistent with greater proteolytic activity in the absence of curcuminoid inhibition [52]. L-Glutamate is an important neuroactive and metabolic amino acid [54], while L-norvaline, a branched-chain amino acid analogue, has shown hepatoprotective properties but potential cytotoxicity at high concentrations [55]. The overall metabolite differences between TEM and CHM fermentations warrant further investigation as indicators of the distinct colonic environments created by curcuminoid-loaded versus empty microparticles.

### 3.7. Limitations and Future Directions

This study has several limitations. The fecal fermentation model, while a validated surrogate for colonic fermentation, does not replicate the dynamic flow, sequential enzymatic digestion, or mucosal–luminal interactions of the intact gastrointestinal tract [56,57]. The curcuminoid concentration used in fermentation (7 mM) may exceed physiologically achievable colonic concentrations after oral TEM administration, potentially overstating the antimicrobial effects on microbiota. In vivo pharmacokinetic studies are required to establish intestinal curcuminoid concentrations achieved after TEM capsule administration and to correlate these with microbiota changes in a clinical setting. Long-term stability data, human pharmacokinetic studies, and randomized controlled trials investigating gut microbiota modulation are needed before the clinical translation of TEMs.

## 4. Materials and Methods

### 4.1. Materials

Curcumin from *Curcuma longa* (CUR), curcumin (≥98% *w*/*w*), bisdemethoxycurcumin demethoxycurcumin, ar-turmerone, indomethacin, lansoprazole, and [3-(4,5-dimethylthiazol-2-yl)-2,5-diphenyltetrazolium bromide] (MTT) were purchased from Sigma-Aldrich, St. Louis, MO, USA. Chitosan, food grade (average viscosity molecular weight of 564 kDa, degree of deacetylation 96%), was purchased from Marine Bio Resources Co., Ltd., Samut Sakhon, Thailand. Mannitol, USP, was purchased from P.C. Drug Center Company Limited, Bangkok, Thailand. Standardized turmeric extract, prepared from powdered turmeric by extraction with 96% ethanol (TEL), was obtained from the Pharmaceutical Laboratory Service Center, Prince of Songkla University, Songkhla, Thailand. Dulbecco’s Modified Eagle Medium (DMEM), Kaighn’s Modification of Ham’s F-12 medium (F-12K), Fetal bovine serum (FBS), penicillin, streptomycin, and 0.25% trypsin-EDTA were purchased from Gibco^®^, Waltham, MA, USA. Peptone water, yeast extract, sodium chloride (NaCl), dipotassium phosphate (K_2_HPO_4_), monopotassium phosphate (KH_2_PO_4_), magnesium sulfate heptahydrate (MgSO_4_·7H_2_0), calcium chloride hexahydrate (CaCl_2_·6H_2_O), sodium bicarbonate (NaHCO_3_), L-cysteine hydrochloride, bile salts, Tween 80, vitamin K, hemin, and resazurin were purchased from Sigma Aldrich.

### 4.2. Preparation and Evaluation of Spray-Dried Turmeric Extract Microparticles (TEMs) for Oral Administration

Spray-dried turmeric extract microparticles (TEMs) were prepared using a blend of chitosan and mannitol as wall materials to encapsulate standardized turmeric extract (TEL) and characterized following the method described by Yupanqui et al. with slight modifications [18]. The wall material solution was prepared by a chitosan–mannitol mixture at a ratio of 1:3.5 in a 1% (*v*/*v*) acetic acid solution. Prior to spray-drying, a turmeric extract solution in 96% ethanol was incorporated into the wall material solution and thoroughly dispersed before processing. The final feeding mixture (5 kg/batch) maintained a curcuminoids:chitosan:mannitol weight ratio of 2:1:3.5, resulting in a total solid content of 7% in the dispersed medium. Chitosan-based microparticles without TEL (CHMs) were prepared as a control. The microparticles were produced using a Pilotech YC-015A Mini inert loop spray dryer (Shanghai Pilotech Instrument & Equipment Co. Ltd., Shanghai, China) equipped with a standard 0.7 mm nozzle. The process was conducted at a dispersion feed rate of 15 mL/min, with inlet temperatures set at 150 °C, an outlet temperature set at 100 °C, an atomizing pressure of 0.1 MPa and an air blower frequency of 35 Hz. Finally, four independent batches were produced, pooled together, and stored in aluminum foil bags for further experiments.

The content of total curcuminoids in TEMs was determined by measuring absorbance at 420 nm using UV–Vis spectrophotometry with curcumin as the reference standard, following the monograph on turmeric extract [17]. Individual curcuminoids, including curcumin, demethoxycurcumin, and bisdemethoxycurcumin, as well as ar-turmerone, served as a biomarker representing the essential oil content; the samples were quantified by high-performance liquid chromatography (HPLC) as previously described [18]. Briefly, samples were extracted with methanol, sonicated for 30 min, and centrifuged at 6000 rpm for 10 min. The resulting supernatants were collected and filtered using a 0.20 µm Whatman membrane filter (Cytiva, Buckinghamshire, UK). HPLC analysis was carried out on a Shimadzu LC-20AD (Shimadzu, Tokyo, Japan) system utilizing an Inertsil^®^ ODS-3 C18 column (4.00 mm × 3.0 mm, 5 µm) protected by a Phenomenex security guard^®^ ODS. The mobile phase consisted of (A) 0.1% (*v*/*v*) aqueous formic acid and (B) acetonitrile, run under gradient conditions: 0–17 min, 50% B (isocratic); 17–28 min, 50–100% B (linear); and 28–35 min, 100% B (isocratic). Separation was performed at 40 °C, with a flow rate of 1.0 mL/min, an injection volume of 20 µL, and UV detection at 254 nm. Disintegration time, moisture content and microbial attributes, including total aerobic microbial count, total yeast and mold count, bile-tolerant Gram-negative bacteria, *Salmonella* species, *Escherichia coli*, *Staphylococcus aureus*, and *Clostridium* species, were assessed as described by the Thai Herbal Pharmacopoeia (THP) [17]. To determine disintegration, one capsule containing TEMs (equivalent to 50 mg curcuminoids) was placed into each of the six tubes of the basket-rack assembly in the disintegration apparatus, and a standard plastic disc was placed on top of each capsule. The apparatus was operated using purified water maintained at 37 ± 2 °C as the immersion medium. The disintegration time for each capsule was recorded at the exact moment the capsule was completely dissolved or dispersed, leaving no remaining residue on the tube’s screen except for fragments of the capsule shell. All six capsules successfully disintegrated within the specified 30 min limit. Heavy metals such as arsenic, cadmium, and lead were analyzed using an Inductively Coupled Plasma Mass Spectrometer (Perkin Elmer, NexION2000, Waltham, MA, USA), while mercury levels were measured by the Direct Mercury Analyzer (NIC MA3000, Takatsuki-shi, Japan).

Hard gelatin capsules No. 0 containing TEMs equivalent to 50 mg of curcuminoids per capsule were prepared and evaluated for physicochemical properties and microbial attributes in accordance with the THP [17]. The TEM capsules were packed in airtight white polyethylene containers and stored at 25 °C and 30 °C with 75% relative humidity for 12 months to assess their stability in accordance with the THP monograph for turmeric extract capsules.

### 4.3. Dissolution Studies

The dissolution of TEMs, TEL, and CUR were examined following the method of Mittraparp-arthorn et al., with some modifications [5]. The USP Dissolution Apparatus II paddle method at a rotation speed of 100 rpm was used. For the experiments, samples containing the equivalent of 50 mg of curcuminoids were immersed in 900 mL of 0.1 M hydrochloric acid solution with 0.05% *w*/*v* polysorbate 80, prepared and used within 12 h and maintained at 37 ± 0.5 °C. At 60 min, 15 mL of samples was withdrawn and filtered through a 0.45 µm filter. The concentration of total curcuminoids in the samples was determined as previously described.

### 4.4. Cytotoxicity and Permeation Studies Using Human Colon Adenocarcinoma (HT-29) Cells

#### 4.4.1. Cell Culture

A human colon adenocarcinoma cell line (HT-29, ATCC HTB-38, Manassas, VA, USA) was cultured in McCoy’s medium containing 10% FBS and antibiotics, 100 U penicillin and 100 U/mL streptomycin, under 5% *v*/*v* CO_2_ at 37 °C. The media were changed every alternate day. When the cells reached confluence, they were harvested using 0.25% trypsin-EDTA, followed by the addition of fresh culture medium to create a new single cell suspension for further incubation.

#### 4.4.2. Sample Preparations

Samples were prepared by dissolution in polyethylene glycol 400 and sonicated for 30 min. The solution was two-fold diluted with cultured medium to obtain a range of concentrations at 7–125 µg/mL.

#### 4.4.3. MTT Assay

The cytotoxicity of samples was evaluated using the MTT assay. Cells were seeded at a density of 1 × 10^5^ cells/well in a 96-well plate (100 µL). After 24 h of incubation at 37 °C in a humidified atmosphere containing 5% *v*/*v* carbon dioxide, the medium in a 96-well plate was replaced with 100 µL of the samples (0–125 µg/mL). Subsequently, the plates were incubated for 24 h. Cytotoxicity was determined using the MTT colorimetric method. The cells were incubated with 80 µL of fresh culture medium and 20 µL of MTT solution at 37 °C in a humidified atmosphere containing 5% CO_2_ for 4 h. After incubation, the MTT-containing medium was removed, and 100 µL of DMSO was added to dissolve the formazan crystals. Absorbance was then measured at 570 nm using a microplate reader (SPECTROstar^®^ Nano, BMG LABTECH, Ortenberg, Germany). A sample was considered cytotoxic if the cell density of the group exposed to the sample was less than 80% when compared to the cell density of the control group.

#### 4.4.4. Permeability Studies

The permeability study was conducted using the apical-to-basolateral (A-to-B) direction at 37 °C with HT-29. HT-29 cell lines at a concentration of 1 × 10^6^ cells/well were seeded in the upper chamber (apical) of the transwell insert. The lower chamber (basolateral) was filled with 1.5 mL of cell culture medium. The culture plate was incubated at 37 °C under 5% CO_2_ for 14 days, replacing the medium in the upper and lower chamber every 3–4 days. Transport experiments were conducted 15 days after seeding. The TEER across the cell monolayers was monitored using a Millicell-ERS in order to assess cell monolayer integrity and the monolayers considered appropriate for the experiment when the TEER values were typically above 200 Ωcm^2^. Prior to transport studies, the medium was removed from all apical and basolateral compartments. One milliliter of sample at a concentration of 15 µg/mL in fresh medium was added into the apical chamber, and 1.5 mL of fresh medium was added into the basolateral chamber. The culture plate was incubated at 37 °C under 5% *v*/*v* CO_2_, and samples were collected from the basolateral chamber (500 μL) at 15, 30, 45, 60, 120 and 180 min. The collected samples were prepared for analysis of biomarkers by mixing 500 µL of fluid and 2 mL of ethyl acetate and vortexed for 2 min. After triplicate extraction, supernatants were evaporated to dryness and resuspended in 200 μL methanol, vortexed for 1 min and transferred to vials. The samples were analyzed for individual curcuminoids and ar-turmerone by HPLC and total curcuminoids by spectroscopy, as indicated in Section 4.2. Apparent permeability (P_app_; in cm/s) was calculated using Equation (1) [28].

(1) $$P_{app}=\frac{dQ}{dt}\times \frac{1}{area\times initial\;concentration}$$

### 4.5. Cytotoxicity and Migration in AGS Gastric Cancer Cells

The human stomach adenocarcinoma cell line (AGS, ATCC CRL-1739, Manassas, VA, USA) was cultured in Kaighn’s Modification of Ham’s F-12 medium (F-12K, Gibco^®^, USA) containing 10% fetal bovine serum (FBS, Gibco^®^, USA) and antibiotics (100 U penicillin and 100 U/mL streptomycin, Gibco^®^, USA) under 5% CO_2_ at 37 °C.

The cytotoxicity of the samples was assessed using the MTT assay, as previously described. The scratch assay was performed to evaluate the cell migration ability of AGS gastric cancer cells, following the procedure described by Wang et al., with some modifications [58]. In brief, AGS cells were seeded at a density of 1 × 10^6^ cells per well in a 12-well plate and incubated for 24 h at 37 °C in a humidified incubator containing 5% CO_2_ to allow cell attachment. A straight scratch was then made in the confluent cell monolayer using a 200 µL pipette tip. Fresh medium or non-toxic concentrations of the test samples (2 mL) were added to each well and further incubated at 37 °C. After 24 and 48 h, the wound areas were imaged using a microphotograph (Olympus CK2, Tokyo, Japan). The remaining wound areas were quantified using ImageJ software (version 1.54).

### 4.6. Cytotoxicity and Inhibition of Nitric Oxide Production in Macrophage RAW 264.7 Cells

A mouse monocyte/macrophage cell line (RAW 264.7, ATCC TIB-71, Manassas, VA, USA) was cultured in Dulbecco’s Modified Eagle Medium (DMEM, Gibco^®^, USA) supplemented with 10% fetal bovine serum (FBS, Gibco^®^, USA) and 100 U/mL penicillin/streptomycin (Gibco^®^, USA). Cells were incubated at 37 °C in a 5% *v*/*v* CO_2_ incubator.

Cytotoxicity was determined using the MTT assay according to established procedures. The inhibitory effect on nitric oxide (NO) production of the microparticles was studied using the macrophage RAW 264.7 cell line, a predominant producer of cytokines. In brief, macrophage RAW 264.7 cells were seeded at a density of 1 × 10^5^ cells/well in a 96-well plate (100 µL). After 2 h of incubation, the medium in a 96-well plate was replaced with fresh medium containing 1 µg/mL of lipopolysaccharide (100 µL), along with 100 µL of samples at concentrations that showed no toxicity. Subsequently, the plates were incubated for 24 h. Nitric oxide production was evaluated by measuring the accumulation of nitrite in the culture supernatant using the Griess reagent at a 1:1 ratio. The concentration of nitric oxide production was measured at 540 nm using a microplate reader. The inhibition of nitric oxide production was calculated according to Equation (2).

(2) $$Inhibition\;of\;nitric\;oxide\;production\left(\%\right)=\left\lfloor \frac{A-B}{A-C}\right\rfloor \times 100$$

The value represents the nitrite concentration (μM) in A: LPS (+), sample (−); B: LPS (+), sample (+); C: LPS (−), sample (−).

### 4.7. Assessment of Gut Microbiota Modulation via In Vitro Fecal Fermentation

#### 4.7.1. Preparation of Fecal Slurry

Fresh feces were collected from five healthy volunteers with a body mass index in the range of 18.50–24.90 kg/m^2^. These volunteers had not consumed prebiotics, probiotics, or antibiotics and had not been diagnosed with any gastrointestinal tract-related diseases for 3 months prior to fecal collection. The fresh feces from each volunteer were placed in plastic jars and stored at a temperature of 4–8 °C. Fecal samples from all 5 people were then mixed together to represent all large groups of microorganisms. Finally, 10 g of the mixture was weighed and mixed with 0.1 M phosphate-buffered saline, pH 7.4, to obtain a 10% (*w*/*v*) fecal slurry. The slurry was homogenized in a blender for 2 min, and the liquid portion was collected after passing through a double layer Stomacher^®^ bag in an anaerobic cabinet [57].

#### 4.7.2. Gut Fermentation in pH-Controlled Batch Culture

One fermenter vessel was prepared for each group (TEMs and CHMs) for microbiota and metabolite analyses. The gut fermentation of the samples was conducted in a pH-controlled batch culture. The composition of the sterilized basal medium included peptone water (2 g/L), yeast extract (2 g/L), NaCl (0.1 g/L), NaHCO_3_ (2 g/L), cysteine⋅HCl (0.5 g/L), K_2_HPO_4_ (0.04 g/L), KH_2_PO_4_ (0.04 g/L), bile salts (0.05 g/L), CaCl_2_·6H_2_O (2 g/L), hemin (0.05 g/L), MgSO_4_·7H_2_O (0.01 g/L), Tween 80 (2 mL/L), vitamin K (10 μL/L), and resazurin (4 mg/L). The sterilized basal medium (90 mL) was poured into each sterilized vessel, which was connected to a circulating water bath set at 37 °C and sparged with O_2_-free N_2_ gas overnight to establish anaerobic conditions prior to the addition of fecal slurry and samples. The pH was maintained at 6.8 ± 0.1 using pH controllers with 0.1 M NaOH or 0.1 M HCl.

The following day, 10 mL of freshly prepared fecal slurry was added to each vessel. Subsequently, 1 g of each sample (TEMs or CHMs) was added to each vessel to achieve a final concentration of 1% (*w*/*v*). A final vessel containing the basal medium and the fecal slurry, without the sample, served as a negative control. The final volume in each vessel was 100 mL. Samples (10 mL) were collected from each vessel at 0, 12, 24 and 48 h for enumeration and metabolic analysis of gut microbiota.

#### 4.7.3. Analysis of Gut Microbiota

The enumeration of gut microbiota was conducted through sequencing of the 16s rRNA gene utilizing Next Generation Sequencing (NGS) technology, specifically employing Illumina platforms. Experimental results were analyzed and interpreted using QIIME2 software (Version QIIME2-202006). Taxonomic compositions at the phylum and genus levels were graphically represented using bar plots and Venn diagrams to illustrate the abundance of amplicon sequence variants (ASVs) detected in the samples. The impact of microparticles on the modulation of gut microbiota, including beneficial genera such as *Bifidobacterium* and *Lactobacillus*, as well as pathogenic species like *Clostridium*, *Bacteroides*, and *Enterobacterium*, were assessed by comparing their abundance before and after incubation in the batch culture.

#### 4.7.4. Analysis of Short-Chain Fatty Acids (SCFAs)

The quantification of short-chain fatty acids (SCFAs), focusing on acetate, propionate, and butyrate, was performed using gas chromatography with a flame ionization detector (GC-FID) [59]. Briefly, the fermented samples from the batch culture were filtered using a 0.22 μm membrane filter. For analysis, 1 μL of the filtered sample was injected into an Agilent HP 6890 GC (Agilent, Santa Clara, CA, USA). Helium was used as the carrier gas at a flow rate of 1.5 mL/min, and separation was carried out on a capillary column (HP 19091 N-113, Agilent, Santa Clara, CA, USA) with a length of 30 cm, a diameter of 320 µm, and a thickness of 0.25 μm. The oven temperature was set to 60 °C for 1 min, ramped up to 100 °C at a rate of 4 °C/min, and maintained for 2 min. Subsequently, it was ramped to 230 °C at 15 °C/min for 5 min. The flow rates of hydrogen, air and nitrogen (the makeup gas) in the FID were 30, 300 and 25 mL/min, respectively. The FID temperature was set to 250 °C, and the total run time for each analysis was 25.67 min. The equilibrium and post times were 1 min each, with a post temperature of 240 °C. Concentrations of acetic acid, propionic acid, and butyric acid were determined by comparison with the calibration standards of each SCFA. SCFA metabolic analyses were performed triplicate at different times for the collected samples.

#### 4.7.5. Analysis of Polyphenol Metabolites

Polyphenol metabolites were analyzed using a liquid chromatography quadrupole time-of-flight mass spectrometer (LC-QTOF-MS). The analysis began by filtering 1 mL of the fermented sample through 0.22 μm nylon membrane syringe filters into a 1 mL LC auto-sampler vial. The optimal conditions were identified in accordance with the specifications of the LC-QTOF-MS (Agilent Technologies, Santa Clara, CA, USA). The LC-QTOF-MS was equipped with a diode array detector and symmetry C18 column (150 mm × 2.1 mm, 1.8 µm particle size), used for tentative identification. Ionization was performed in negative electrospray (ESI) mode. The identification of polyphenol metabolites in the fermented sample was performed in negative ion mode, with different concentrations of acetonitrile and water as the mobile phase. The MS condition was detected between 50 and 1200 *m*/*z*. The desolvation and column temperatures were maintained at 375 °C and 30 °C, respectively. The flow rate (0.2 mL/min) and injection volume (2 µL) were set for system operation. The capillary voltage and desolvation gas flow rate were set at 4.0 kV and 13 L/min, respectively. MassHunter METLIN Metabolite software (version 5.0), with a scientific library, was used to identify the polyphenol metabolites.

### 4.8. Data Analysis

Statistical analyses were performed using SPSS version 22 (SPSS Inc., Chicago, IL, USA). Data (*n* = 3 or 4) are expressed as mean ± standard deviation (SD). Continuous variables between two groups were compared using an independent-sample *t*-test, whereas multi-group comparisons were evaluated using a one-way analysis of variance (ANOVA) followed by Tukey’s post hoc honest significant difference (HSD) test. Statistical significance was defined as *p* < 0.05. For gut microbial profiling, the obtained amplicon sequence variants (ASVs) were annotated to determine their corresponding taxonomic classifications and abundance distributions. Alpha-diversity metrics (including the Chao1 and Shannon indices) were calculated using R software (version 4.2.3) to evaluate microbial community richness and evenness within individual samples.

## 5. Conclusions

This study successfully developed TEMs as a cost-effective oral delivery system. Utilizing an extract of whole turmeric—which retains both curcuminoids and turmeric oil—combined with a chitosan carrier offers distinct manufacturing advantages over purified curcumin, including reduced production costs and shortened processing times. The resulting orange micronized powder exhibited favorable spherical morphology, and capsule formulation complied with regulatory standards for oral herbal products.

Physicochemically, the TEM formulation achieved an approximate 40-fold and 5-fold increase in curcuminoid dissolution within simulated gastric fluid compared to unencapsulated extract and purified curcumin, respectively. However, while TEMs demonstrated effective transepithelial transport, the intestinal permeability in HT-29 cell monolayers was comparable to that of the unencapsulated extract. This indicates that while the chitosan carrier significantly drives dissolution, it provides limited enhancement for direct cellular permeability.

Biologically, TEMs demonstrated potent, dose-dependent therapeutic effects, yielding a two-fold higher inhibition of gastric cancer cell growth than curcumin and a significant suppression of inflammatory nitric oxide production compared to the unencapsulated extract. Furthermore, fecal fermentation profiles revealed that TEMs dynamically modulate the gut microbiota. Although a transient increase in potentially harmful bacteria occurred during early fermentation phases, these populations were successfully suppressed at later stages, culminating in a metabolic profile dominated by acetate. The absence of detectable curcuminoid-derived polyphenol metabolites under simulated colonic conditions suggests that the parent compounds or alternative metabolic pathways drive these microbial shifts.

Taken together, these findings establish TEMs as a highly promising, scalable, and multifunctional therapeutic strategy for the management of gastrointestinal diseases. Future research should focus on in vivo evaluations to validate these delivery dynamics and confirm long-term safety profiles.

## Figures and Tables

**Table 1:** Biomarker content in samples (mean ± SD, *n* = 3 independent replicates).

Code	% *w*/*w* Biomarker Content				
	Cum ^a^	Dem ^a^	Bis ^a^	Ar-Turmerone ^a^	Curcuminoids ^b^
TEMs	13.09 ± 0.31	5.17 ± 0.03	5.88 ± 0.18	6.51 ± 0.04	24.64 ± 0.36
TEL	6.13 ± 0.07	4.97 ± 0.05	8.77 ± 0.12	9.37 ± 0.02	20.73 ± 0.78
CUR	71.73 ± 0.32	17.38 ± 0.04	7.73 ± 0.02	Not detected	102.05 ± 1.23

Note: TEMs = Turmeric extract microparticles, TEL = Turmeric extract in liquid form, CUR = Curcumin from *Curcuma longa*, Cum = Curcumin, Dem = Demethoxycurcumin, Bis = Bisdemethoxycurcumin. Method of analysis: ^a^ = High Performance Liquid Chromatography, ^b^ = Spectroscopy.

**Table 2:** Certificate of analysis of finished product. Name: Turmeric Extract Microparticle Capsule. Each capsule contains turmeric extract microparticles equivalent to 50 mg curcuminoids, calculated as curcumin, and ethanol 96% as solvent.

Test	Specification	Result
Appearance	Hard gelatin capsule No. 0, containing yellow to brownish orange powder	Conform
Heavy Metal Contamination		
Arsenic	Less than 5 ppm	<0.05 ppm
Cadmium	Less than 0.3 ppm	Not detected
Lead	Less than 10 ppm	0.13 ppm
Mercury	Less than 0.5 ppm	Not detected
Microbial Contamination		
Total Aerobic Microbial Count	Not exceed 2 × 10^4^ cfu/g	<10 cfu/g
Total Yeast and Mold Count	Not exceed 2 × 10^2^ cfu/g	<10 cfu/g
Bile-tolerant Gram-negative Bacteria	Not exceed 10^2^ cfu/g	<10 cfu/g
*Salmonella* spp.	Absence	Absence
*Escherichia coli*	Absence	Absence
*Clostridium* spp.	Absence	Absence
Weight Variation	Not more than 10% of the average weight	4.18%
Disintegration Time	Within 30 min	1.76 min
Potency	Not less than 90% of the labeled amount	108.88%
Loss on Drying	Not more than 7.0% *w*/*w*	1.92%

**Table 3:** Percentage of curcuminoid release in simulated gastric fluid at 60 min and appearance permeability in HT-29 model during 180 min.

Samples	%Release 60 min	P_app_ (10^−6^ cm/s)				
		30 min	45 min	60 min	120 min	180 min
TEMs	36.18 ± 1.93 *	5.95 ± 2.53	7.76 ± 2.59	8.19 ± 1.72 *	10.03 ± 2.52 *	1.78 ± 0.36 *
TEL	0.87 ± 0.19 *	3.09 ± 1.06	4.71 ± 1.51 *	5.45 ± 0.65 *	6.86 ± 0.83 *	1.41 ± 0.18 *
CUR	7.58 ± 0.37	9.17 ± 3.09	11.93 ± 2.65	14.22 ± 2.47	17.72 ± 1.88	3.04 ± 0.14

Note: P_app_ = Apparent permeability, calculated using total curcuminoid content in the basolateral side at the end of the transport experiments after 180 min. TEMs = Turmeric extract microparticles, TEL = Turmeric extract in liquid form, CUR = Curcumin from *Curcuma longa*. * = *p*-value < 0.05 when compared with CUR. Data expressed as mean ± SD, *n* = 3 independent experiments.

**Table 4:** The production of short-chain fatty acids during fermentation (mean ± SD, *n* = 3 technical replicates from a representative experiment).

Duration	Short-Chain Fatty Acids (mM)		
	Acetic Acid	Propionic Acid	Butyric Acid
Before fermentation	2.38 ± 0.18	0.18 ± 0.00	0.53 ± 0.04
Turmeric extract microparticles			
12 h	5.15 ± 0.13 *^c^	0.18 ± 0.00 ^b^	0.34 ± 0.04 *^a,b^
24 h	8.59 ± 0.20 *^a^	0.29 ± 0.03 *^a^	0.40 ± 0.05 *^a^
48 h	6.91 ± 0.15 *^b^	0.18 ± 0.00 ^b^	0.29 ± 0.00 *^b^
Chitosan-based microparticles			
12 h	9.57 ± 0.07 *^b^	0.18 ± 0.00	0.29 ± 0.01 *
24 h	12.10 ± 0.45 *^a^	0.18 ± 0.00	0.30 ± 0.00 *
48 h	12.46 ± 0.11 *^a^	0.18 ± 0.00	0.28 ± 0.01 *

Note: *: *p* < 0.05 compared to before fermentation, ^a,b,c^: *p* < 0.05 compared to each duration in the same group.

**Table 5:** Related polyphenol metabolites during fermentation.

Compounds	% Abundance				
	Before Fermentation	TEMs 24 h	TEMs 48 h	CHMs 24 h	CHMs 48 h
Lysine	-	11.86	-	23.48	-
D-Ornithine	-	20.46	-	38.16	-
L-Glutamate	10.82	8.27	-	9.42	-
γ-Aminobutryic acid	9.72	8.88	-	16.34	-
L-Norvaline	4.38	-	-	12.6	-
D-Fructose	-	4.2	-	-	-
L-Valine	-	10.43	-	-	-
(7E)-2-Acetyl-2,3,4,5-tetrahydrooxonine-6,9-dione	-	8.65	-	-	-
Formylisoglutamine	-	-	17.3	-	-
Butoxyacetic acid	-	-	38.8	-	42.13

## Data Availability

The datasets generated and/or analyzed during the current study are not publicly available due to ethical and institutional restrictions related to patient confidentiality. Data are available from the corresponding author on reasonable request and with approval from the relevant institutional review board.

## Abbreviations

TEM: turmeric extract microparticle; SEM: Scanning electron microscopy; CUR: curcumin from *Curcuma longa*; TEL: turmeric extract; HPLC: high-performance liquid chromatography; SGF: simulated gastric fluid; SIF: simulated intestinal fluid; P_app_: apparent permeability coefficient; IC50: half-maximal inhibitory concentration; SCFA: short-chain fatty acid; MTT: 3-(4,5-dimethylthiazol-2-yl)-2,5-diphenyltetrazolium bromide.
